# Flow Chemistry in Contemporary Chemical Sciences: A Real Variety of Its Applications

**DOI:** 10.3390/molecules25061434

**Published:** 2020-03-21

**Authors:** Marek Trojanowicz

**Affiliations:** 1 Laboratory of Nuclear Analytical Methods, Institute of Nuclear Chemistry and Technology, Dorodna 16, 03–195 Warsaw, Poland; trojan@chem.uw.edu.pl; 2 Department of Chemistry, University of Warsaw, Pasteura 1, 02–093 Warsaw, Poland

**Keywords:** flow analysis, flow synthesis, flow reactors, flow-injection analysis

## Abstract

Flow chemistry is an area of contemporary chemistry exploiting the hydrodynamic conditions of flowing liquids to provide particular environments for chemical reactions. These particular conditions of enhanced and strictly regulated transport of reagents, improved interface contacts, intensification of heat transfer, and safe operation with hazardous chemicals can be utilized in chemical synthesis, both for mechanization and automation of analytical procedures, and for the investigation of the kinetics of ultrafast reactions. Such methods are developed for more than half a century. In the field of chemical synthesis, they are used mostly in pharmaceutical chemistry for efficient syntheses of small amounts of active substances. In analytical chemistry, flow measuring systems are designed for environmental applications and industrial monitoring, as well as medical and pharmaceutical analysis, providing essential enhancement of the yield of analyses and precision of analytical determinations. The main concept of this review is to show the overlapping of development trends in the design of instrumentation and various ways of the utilization of specificity of chemical operations under flow conditions, especially for synthetic and analytical purposes, with a simultaneous presentation of the still rather limited correspondence between these two main areas of flow chemistry.

## 1. Introduction

Monitoring and controlling the progress in the course of a given chemical reaction is a fundamental issue in various applications of chemical science. This concerns fundamental investigations into both the composition and the properties of various materials, as well as studies on various phenomena and processes occurring on micro- and macro-scales in the natural environment and in living organisms. It also concerns the optimization of various technological processes involving specific chemical reactions. As indicated by the progress in different areas of chemical science achieved over the past century (or more), one of the contributing factors in the yield of chemical reactions is the movement of reagents depending on various mechanisms. In both laboratory and industrial practices, the most commonly employed processes are those under conditions of the forced flow of reagents. Changes in the transport rate of reagents under flow conditions can be utilized in the physico-chemical examination of the kinetics of the reaction, as well as for the improvement of the efficiency of different steps in analytical procedures or for carrying out chemical synthesis with favorable yields.

In spite of the large number of various applications of carrying chemical reactions under flow conditions, the term “flow chemistry” can only recently be found in the chemical literature, and it is used almost exclusively for the description of chemical syntheses carried out under flow conditions. The search of literature databases indicated its first uses in the 1970s in various fields such as modeling of chemical laser operations [1], fabrication of materials for the nuclear industry [2], transport of pollutants associated with irrigation [3], or the aerothermodynamic analysis of a stardust sample return capsule in the National Aeronautics and Space Administration (NASA) mission [4]. First examples of the use of the term “flow chemistry” in chemical synthesis or analytical fields were found at the turn of 1990s and 2000s in work on the pulsed generation of concentration profiles in flow analysis [5] and in the description of continuous-flow microreactors with fluid propulsion achieved by magnetohydrodynamic actuation, which was employed for the amplification of DNA through the polymerase chain reaction [6]. This can be considered to be an analytical device; however, it simultaneously works via the synthesis of the desired product.

In the case of papers dealing with syntheses under flow conditions, so far generally considered in the present chemical literature as the only field of flow chemistry, the term “flow chemistry” started to be used from the middle of the 2000s onward [7]. The term “flow synthesis”, in turn, is commonly used in papers on organic synthesis since the 1970s [8].

The volume of published papers on widely recognized flow chemistry, including not only chemical synthesis but also flow analysis, fundamental physico-chemical investigations under flow conditions, or flow reactors in industrial applications, can be estimated to be 30 to 40 thousand papers in both scientific and technical journals. It should be taken into account that a very arbitrary selection of published works had to be made for the present review. Therefore, for instance, certain areas of measurements or studies under flow conditions are not included, for example, analytical process monitoring by dedicated industrial instrumentation or industrial processes carried out on a technological scale in flow reactors. This should be mentioned in order to not restrict the term “flow chemistry” in the context of modern chemical science and, simultaneously, to point out the importance of this field.

The intention of the author of this review is to present, for the first time in the literature, various fields of modern chemistry which should be considered within *flow chemistry*. A special emphasis is focused on the presentation of the development and chronology of inventions of numerous physico-chemical operations and appropriate instrumental devices, which are widely employed in both flow synthesis and flow analysis.

## 2. Milestones in the Development of Various Areas of Flow Chemistry

Although it is hard to underestimate the benefits of using numerous literature databases, tracing the evolution of various methodologies of conducting measurements or carrying out chemical syntheses under flow conditions is a very challenging task. The results of different applications of flow chemistry are distributed very broadly in hundreds of chemical journals and they are not always reported in the available databases. The general intention of preparing this section of the review is to present (at least roughly) the chronology of the development of the selected areas of flow chemistry.

### 2.1. Flow Analysis

It seems that the first chemical phenomenon to be observed under flow conditions and employed for analytical purposes was the separation of a mixture of chemical compounds on a flow-through column packed with a solid sorbent, which initiated the development of chromatographic methods. This is commonly attributed to Cwett’s works conducted at the University of Warsaw at the beginning of the 20th century [9,10], although a similar observation was published also earlier [11]. It is necessary, however, to admit that, regardless of the similarity of both operations, the commonly used term “flow analysis” is rather associated with the much later invention of continuous-flow analysis with the segmentation of flowing stream by Skeggs in the middle of the 1950s [12,13], where the segmentation of a flowing stream with air bubbles was essential for limiting the analyte dispersion along the tubing. The developed pioneering system consisted of several instrumental flow-through modules, which allowed performing different physico-chemical operations for the clinical determination of urea in blood with photometric detection in a mechanized manner. It means then that conventional flow analysis follows instrumental set-ups (schematically presented as manifolds), where determinations with different detection techniques can be carried out with various operations of on-line sample processing, with or without the segmentation of the flowing liquid. This understanding of flow analysis is confirmed by a large number of published books and review papers in various journals [14]. This also means that, despite involving flowing conditions, chromatographic methods, electrophoretic methods, mass spectrometry, or atomic spectrometry methods, where the flow of analytes from the sample introduction to the detector takes place, do not fall into the flow analysis category. The concept of constructing a flow analyzer with a segmented stream was used to develop many set-ups for numerous applications in various areas of analytical chemistry [15], as well as for commercial instruments by specialized manufacturers. 

In further modification of that concept, the segmentation of a flowing liquid was eliminated from the measurement system [16], a very small sample volume (20–200 μL) was used [17], and a transient signal was used (as an analytical one) instead of steady signal values recorded in the segmented flow analyzers. An extraordinary impetus for the further development of that version of flow analysis called flow-injection analysis (FIA) was given by a series of papers published in the middle of the 1970s by Ruzicka and Hansen [18,19,20], as well as some parallel ones by Stewart et al. [21,22]. Fast development of that methodology resulted in the availability of numerous commercial instruments [23], as well as further development of the modified versions of FIA such as sequential injection analysis (SIA) [24] or lab-on-valve (LOV) systems, which integrated the injection modules with detection and some operations of sample treatment on a renewable bed of solid sorbents [25].

Already at the end of the 1970s pioneering microfluidic systems were designed and produced on silicon wafers, firstly employed as a capillary column in gas chromatography [26]. Then, since the beginning of the 1990s, their first applications in flow analysis were developed, e.g., in hyphenation with the surface plasmon resonance analyzer for the determination of immunoglobulins [27], or in a miniaturized system with detections by solid-state electrochemical sensors or small-volume optical detectors [28]. The currently observed development of measuring systems involves further miniaturization and instrumental integration of hydraulic, detecting, and sample processing operations. This is also associated with tapping into current achievements of nanotechnology and new technologies for the transmission and processing of measured signals.

### 2.2. Flow Synthesis

Organic chemistry is an exceptionally broad area of modern chemistry embracing fundamental investigations of mechanisms of reactions, identification of natural compounds, and optimization of laboratory syntheses, as well as their scaling up to a technological level. Utilizing flow conditions for carrying out chemical syntheses is a very important part of the development of flow chemistry. Although, as already mentioned, the term “flow chemistry” was used only recently, i.e., in the last two decades, the real beginnings of this methodology were much earlier. According to the Web of Science database, the first contribution to flow synthesis was a short report on the use of a flow reactor with a phosphoric acid catalyst on silica gel for dehydration of diethylcarbinol, published in 1932 [29]. An evident increased interest in the use of flow reactors, especially those with an immobilized catalyst, was noted no earlier than the 1960s [30,31]. Numerical methods were employed to evaluate the participation of diffusion, the convection of a reactant, and the kinetics of homogeneous and heterogeneous reactions in catalytic tubular reactors [30], while experimentally diffusional and chemical effects were examined for fast reactions of cyclopropane in flow reactors [32]. Flow synthesis in an open flow-system was reported for producing layers of gallium phosphide for some electronic applications [33]. A continuous-flow salt gradient was utilized in the preparation of polynucleotide–polypeptide complexes [34], while excellent yields in cryptate synthesis could be obtained via a cyclization reaction carried out with efficient mixing in a suitable flow cell [35]. The 1980s brought further development in designing automated systems where the progress in flow synthesis was monitored on-line and was increasingly controlled by the computer, which was reported, e.g., for solid-phase synthesis of peptides [36,37] and oligodeoxyribonucleotides [38]. In the latter case, more than 600 oligomers were produced in approximately 15 min. Alpha-gliadin peptides were synthesized in a continuous-flow set-up under ultrasonic field conditions, which accelerated the coupling reaction [39].

A substantial increase in the number of published papers on various innovations in flow synthesis was observed since the beginning of the 2000s. It seems that this can be essentially attributed to an increasing interest in this technology and its great potential for speeding up drug development by leading manufacturers of pharmaceutical industries [40], as well as by very active research groups from top academic institutions such as, e.g., the University of Cambridge in the United Kingdom (UK) [41], the California Institute of Technology [42], or the Massachusetts Institute of Technology (MIT) [43]. Numerous flow syntheses can be successfully enhanced with microwave activation [41,44], while conventional pumping in the flow systems with piston pumps can be replaced with a magnetohydrodynamic actuator [6] or by the use of electroosmotic flow [45]. Flow syntheses can be also carried out under supercritical conditions, which was first employed when producing narrow-size distribution quantum dots [46]. 

As of the middle of the 2000s, flow syntheses with the use of microfluidics were performed more and more commonly, allowing for the reduction of the amounts of reagents and solvents—a pioneering example of gas–liquid–solid hydrogenation [47] or the production of nano amounts of a ^19^F-labeled imaging probe for positron emission tomography (PET) [42]. Yet another direction in this trend was the use of droplet-based microfluidics, which was demonstrated, for instance, for the synthesis of anisotropic gold nanocrystal dispersions [48].

As it was shown, e.g., in the synthesis of the natural alkaloid called oxomartidine, complete synthesis of natural products can be carried out in a multistep procedure in the system with several packed columns containing immobilized reagents, catalysts, scavengers, or catch-and-release steps [7]. This work initiated intensive development of some other, similarly complex systems, also performed in microreactor networks, e.g., in the multistep synthesis of carbamate [49] or in the continuous-flow synthesis of carboxylic acids with the use of so-called tube-in-tube gas-permeable membrane reactors [50].

The most recent instrumental innovations in flow syntheses are, among others, the use of three-dimensional (3D) printed advanced reactors for Ag nanoparticles [51] or the creation of a robotic system for flow synthesis [52].

### 2.3. Fundamental Physico-Chemical Measurements under Flow Conditions

One of the areas where measurements under flow conditions were applied for decades is the investigation into the kinetics of chemical reactions, especially in the case of fast reactions taking place in solutions. Reports on such measurements can be found in the literature since the 1950s [53], while the stopped-flow methodology for this purpose was reported even earlier [54]. In the latter method, instead of the measurements of concentration changes in the monitored reaction along the length of the reaction tube, the detection system is located in the proximity of the mixing chamber into which the solutions of reagents are forced from the syringes. Kinetic measurements were also carried out in a typical flow analyzer with a segmented stream in studies on peptide bond hydrolysis [55]. Kinetic measurements in a stirred flow reactor for the alkaline bromination of acetone allowed evaluating both reaction orders, as well as determining the reaction-rate constants in a system of consecutive reactions [56].

Contemporary kinetic measurements are carried out with different detection techniques and in different configurations of flow systems. For instance, a successful application of this kind was reported for continuous-flow microfluidic devices with laser-based mid-infrared chemical imaging in studies on fast organometallic chemistry [57], while a microfluidic system—with in situ X-ray and fluorescence detection—was used in studies on hydrogelation kinetics [58]. In a microreactor set-up, kinetic data on exothermic synthesis by Michael addition was examined with fast in-line monitoring by Raman spectroscopy [59]. Then, two recently reported examples described kinetic studies devoted to free-radical chemistry under flow conditions with chemiluminescence detection. Infrared chemiluminescence detection was employed to monitor the kinetics of the reaction of ^•^OH radicals with formamide and chemically activated carbamic acid [60]. The chemiluminescence of a methyl- cypridina luciferin analogue was employed to examine the kinetics of the reaction of superoxide radical ions with dissolved organic matter in a typical flow-injection analysis system [61].

In stopped-flow measurements, where the dead-time of a fraction of a millisecond can be reached in modern instrumentation, various spectral and electrochemical methods are used to investigate the kinetics of very fast reactions in solutions. Absorption or fluorescence spectroscopy are the two most commonly used detection techniques in dedicated commercial instruments. In a very early example of such works, the kinetics of unstable compounds in a biochemical reaction (e.g., the formation of catalase–hydrogen peroxide complex) was reported with spectrophotometric detection in a time span from milliseconds to several minutes [62]. Then, a very recent example was the examination of the kinetics of aroxyl-radical scavenging rates by important lipid-soluble antioxidant α-tocopherol and catechins [63]. An example of some recent stopped-flow kinetic investigations with the use of fluorimetric detection was a study on the binding of biotin and biocytin to avidin and streptavidin, which is broadly used in biotechnology and bioanalysis [64]. Creating a simple manifold that can be attached to a commercial stopped-flow apparatus, allowing kinetic studies to be performed from −12 °C to 45 °C, constitutes an instrumental improvement in such measurements reported in recent years [65]. The sensitivity of kinetic absorption measurements in ultraviolet (UV)–visible wavelengths can be enhanced by the use of optical cavity-based techniques. The reported low-cost experimental set-up providing broadband cavity enhancement of sensitivity by coupling to commercial stopped-flow instruments allows obtaining even 78-fold enhancement for the measurements at 434 nm, which was employed in the investigation of the kinetics of a potassium ferricyanide reaction with sodium ascorbate [66].

Among other spectroscopic detection techniques used under flow conditions, numerous applications in stopped-flow kinetic measurements were reported for circular dichroism (CD). This technique enables simultaneous monitoring of chiro-optical and absorbing transients, which was shown, for instance, in the binding of sulfonamide to bovine carbonic anhydrase [67]. Recently, CD detection is commonly employed to study protein folding kinetics [68], while ultraviolet CD was used to study the folding mechanism of the outer surface protein A. Kinetic measurements in a stopped-flow mode are also used with Fourier-transform infrared (FTIR) detection [69], whereas NMR spectroscopy was reported, for instance, in the examination of metallocene-catalyzed poly- merization of 1-hexene [70].

Electrochemical detections are used much less commonly in detection of stopped-flow measu- rements than in spectroscopy techniques. At the very early stage of the development of stop- ped-flow methodology, conductivity detection and potentiometric pH measurements were employed to study the following reaction: OH^−^ + CO_2_ → HCO_3_^−^ [71]. One recent example showed, e.g., the use of voltammetry in stopped-flow studies on the kinetics of reduction of cop- per-containing enzyme peptidylglycine monooxygenase [72]. The above-mentioned examples clearly show a crucial role of flow measurements in the evaluation of kinetic parameters for many different chemical processes.

### 2.4. Chemical Processing under Flow Conditions for Non-Analytical and Non-Synthetic Purposes

This section of the present review only very briefly highlights the importance of yet another field of investigations and processes which should also be considered as a substantial part of *flow chemistry*. It is based on research studies and processes oriented most commonly toward technological applications in flow reactors, but they are also commonly initiated in laboratory investigations under flow conditions. Moreover, they are usually investigated for final technological applications in environmental protection or the processing of various materials in different branches of industry, including food processing. The examples presented below were randomly and arbitrarily selected to roughly indicate the variety of areas and problems associated with this particular field of flow chemistry. 

As already mentioned, these approaches do not follow the rules of the field of chemical engineering on a technological scale. A vast amount of literature on this subject can be found in technological journals, patents, and specialized books [73,74]; thus, in this section, only some examples from the current literature are provided.

Applications of a flow-through reactor with immobilized α-galactosidase, which was used in the reduction of raffinose concentration in beet sugar molasses [75], or in the application of a flow-through reactor for the photocatalytic decomposition of ethylene applied to control tomato ripening [76], are two examples of employing biotechnology in the food processing field. A dynamic flow approach was reported to examine the leaching of antioxidants from solid foods [77], while, for ore and mineral processing, a reflux flotation flow cell was designed to achieve fast flotation [78]. 

The processing of biomass of various origin, which can be carried out in flow-through reactors, is an important sector of modern biotechnology. For instance, the flash pyrolysis of microalgal or lignocellulose biomass in a flow reactor may lead to the production of bio-oils of a very different composition, depending on the type of an initial substrate and the conditions of pyrolysis [79]. The obtained products can be further employed as phenolic-rich bio-oils, liquid fuels, or fine chemicals. The hydrothermal conversion of a wet waste feedstock in a continuous-flow reactor may result in obtaining liquid or gas final products with acceptable residual organic contamination [80]. The production of bio-crude oil using hydrothermal liquefaction in a continuous-flow reactor was also reported from wastewater microalgae [81].

A particularly large number of research papers on both the investigation and the application of flow-through reactors were devoted to the development of different processes for environmental protection, involving various methods of chemical processing. The biological anaerobic removal of nitrate from wastes was developed with the use of a bioreactor packed with sludge carbonaceous material [82]. In yet another similar method for the biodegradation of 2-chlorophenol, adsorption and biodegradation processes were carried out in a flow-through reactor with suspended biomass on activated carbon [83]. The mechanism of removal included the sorption of a pollutant, its transport by diffusion in a biofilm, and biodegradation by suspended biomass. Just recently, the efficiency rates of bacterial aerobic granular sludge and algal–bacterial granular sludge packed in a 20-L continuous-flow reactor were compared for saline wastewater treatment [84]. Since high salinity enhances algae growth, the second material exhibited slightly higher total nitrogen and phosphorus removal efficiency.

Typical chemical processing methods were also widely investigated for water and wastewater treatment in flow-through reactors. These approaches include, first of all, various photochemical methods, involving (mainly) UV irradiation under different conditions with or without catalysts [85]. For instance, the photocatalytic degradation of bio-resistant dyes was demonstrated in a flow reactor with a TiO_2_ catalyst embedded into a cement matrix and deposited at the bottom [86]. Regarding other methods, the catalytic wet air oxidation of industrial wastewater was carried out in a flow reactor packed with copper nanoparticle-doped and graphitic carbon nanofiber-covered porous carbon beads [87]. A complete chemical oxygen demand (COD) reduction in industrial wastewater was observed under optimized conditions. A significant reduction (50% to 80%) of COD and the removal of numerous inorganic contaminants from drinking water could be obtained with the use of a flow reactor packed with a mixture of anionic and cationic ion-exchange resins [88].

The processes such as sonolysis, electrochemical oxidation, or irradiation with a beam of accelerated electrons can be employed in continuous-flow reactors for the efficient decomposition of organic pollutants in waters and wastewaters. For instance, separate or simultaneous sonolysis and ozonation were examined under flow conditions for the degradation of antibiotics in wastewater [89]. Electroactive pollutants such as residues of different pharmaceuticals can be efficiently removed by electrochemical oxidation, which was demonstrated, e.g., for the residues of the widely used anti-inflammatory drug called naproxen using different porous materials of anode [90]. A flow reactor with a boron-doped diamond anode enabled efficient degradation of azo dyes occurring in synthetic textile effluent [91], whereas an ultra-nanocrystalline, boron-doped diamond anode coated on a niobium substrate, also employed in a flow reactor, was successfully used for the minera- lization of a persistent pollutant perfluorooctanoic acid [92]. An aerosol flow reactor developed for the efficient decomposition of organic pollutants by electron beam irradiation can be mentioned as a final example of different flow reactors investigated for environmental purposes [93].

The above-mentioned examples are only a few randomly selected examples that can be found in thousands of papers published in scientific journals and patents on the use of flow reactors for this field of research activity. It is impossible to provide a more complete review of these applications or to discuss the evolution of their construction, the optimization of their operation, or the broad fields of applications. It is doubtless, however, whether these types of chemical processes should also be considered a part of flow chemistry. It is also impossible not to admit that the processes investigated and optimized in such flow reactors are often utilized in flow systems developed for both analytical and synthetic purposes, contributing to the final results of their functioning.

## 3. Similarities in Development Trends in Flow Analysis and Flow Chemistry

Each of the four above-mentioned areas of flow chemistry underwent several decades of development, and, behind each, there is vast subject literature and numerous applications. It seems, however, that, in terms of the scale of reported processes, the choice of the employed physico-chemical unit operations, and the adoption of similar instrumental constructions, the closest similarity can be found between flow analysis and laboratory flow synthesis. Although this was already pointed out in an earlier review [94], as well as in the Website review [95], there is still almost a complete lack of interrelation between scientists contributing to each field, i.e., organic chemists working on flow synthesis and analysts working on flow analysis, which inclined us to present the development trends in these two areas of flow chemistry once again.

The development/evolution of flow analysis methods takes place in three basic ways. The first one is the instrumental development of detection systems, pumping devices, and on-line sample treatment modules. The second one deals with the development of methodologies of flow measurements and signal processing, while the third one concerns both the search for and the selection of the most suitable chemistry for the most successful determination of particular analytes. As mentioned before, according to the well-established custom, the flow analysis field does not include chromatographic and electrophoretic methods in the further discussion below. 

The development of flow analysis in these three aspects aims at establishing the most efficient instrumentation and procedures, providing the most accurate, precise, and selective determination, while requiring the simplest instrumentation and smallest possible human effort during its routine use. The evolution of detecting devices tends to obtain the best possible accuracy and selectivity, appropriate detectability for particular applications, and the shortest response time. A significant contribution to obtaining satisfactory accuracy and selectivity was brought by the employed on-line pretreatment steps. The evolution of measuring methodologies and their selection for particular determinations is connected to the mode of signal acquisition, its processing into analytical information, and the choice of a calibration method. Moreover, the evolution of “chemistry of analytical determination” involves the interaction of an analyte with a detector or an employed analyte/sample pretreatment method, which provides the best sensitivity of detection with satisfactory selectivity.

To a great extent, similar factors, although with certain shift of accents, seem to be taken into consideration in flow systems for chemical syntheses. Obviously, in synthetic flow systems, the heart of the whole instrumentation is not the detector itself but the flow-through reactor(s). Instead of the sensitivity of detection, the most important factor is the yield of the conducted reaction. Synthesis time, necessary supervision by a human operator, and optimization of both the design and the functioning of different modules for carrying out some intermediate steps (mixing, cooling, heating, etc.) are also of critical importance. It seems that one of the most crucial differences between analytical and synthetic flow systems is the fact that, in the case of the former, a transient signal received from the detectors can be used as a useful analytical piece of information (assuming their satisfactory precision), while, in the case of flow synthesis, a crucial parameter is the completeness of the desired product and its purity, which play a significant role in the scavenging steps of many procedures.

### 3.1. Detectors, Reactors, Manifolds

More than six decades of the development of flow analysis can be found in over 20 thousand publications in various scientific journals. A list of books published on this subject is shown in Table 1 (see Conclusions and Perspectives section), while a large selection of numerous review papers can be found in an earlier review [14]. Such a vast literature provides a selection of examples for an in-depth discussion on the comparison of progress in flow synthesis. The development of very efficient flow-through detectors is a fundamental task for analytical systems. Generally speaking, they should be able to generate a signal of the largest possible sensitivity with very fast response time at the smallest possible dead-volume.

From the very beginning of the development of flow analysis, the greatest attention was given to spectrophotometric absorption detection in the UV–visible region, molecular and electrochemical luminescence, and the application of atomic spectrometers. The evolution of the simple cost-effective construction of detectors is associated with parallel development, provided mostly by the manufacturers of commercial instrumentation and detectors for liquid chromatography. In the case of spectrophotometric detectors, important progress was made in the replacement of simple flow cuvettes with long pathway detectors, utilizing multiple internal reflection [96], in conducting absorption measurements in cuvettes packed with solid sorbents on the optical pathway [97], or in the use of optoelectronic elements as a light source [98], as well as a detector. The simultaneous use of several different light-emitting diodes (LEDs) as a light source allows carrying out multi-analyte detection [99], while a suitable design of the detector cell enables conducting absorptive, fluorometric [100], nephelometric, and turbidimetric detections [101]. The development of common types of electrochemical detectors (thin-layer, wall-jet, wire, cascade, or multi-array detectors) was accompanied by the search of new electrode materials and surface modification of the working electrode surface, especially with biochemical receptors and nanomaterials [102]. The possibility of conducting simultaneous multi-analyte detection (without chromatographic separation) was achieved by appropriate polarization of the working electrode in voltammetric measurements [103], reporting a multi-pulse amperometric detection or the application of various sensors such as a potentiometric sensor with limited selectivity but with appropriate signal processing [104]. An example of such an advanced attempt was a rapid solid-phase fluoroimmunoassay [105]. There are specific instruments for flow analysis; however, as presented below, some flow detectors can be directly incorporated into flow synthesis systems for real-time monitoring of the progress of the conducted reaction.

A different strategy was used in the development of flow reactors for flow synthesis systems. In conventional laboratory systems (mini- or meso-fluidic), made of glass, quartz, or metallic coiled tubes of an internal diameter of a few mm, the volume of a reactor is not very important. In most cases, good heat transfer, simultaneous irradiation (in the case of photochemical reactions) [106], and microwave heating [107] are of bigger importance. A separate group of tools includes microreactors made of stainless steel or perfluorinated polymer tubes, or micro-structured devices [108]. A particular advantage of carrying out flow syntheses in microreactors is using very tiny amounts of substrates and products, which is crucial when using hazardous chemicals [109]. Microfluidic reactors can be manufactured using glass, silica glass, ceramics, or stainless steel by micro- machining. One reactor made of silicon was employed in the above-mentioned set-up with fluid propulsion by alternating current (AC) magnetohydrodynamic actuation [6]. Teflon microreactors were also developed into a “click-system” version, comprising two separate plates of Teflon fabricated by computerized numerical control milling [110]. A particular reported design was a microcapillary reactor made in the form of a fluoropolymer disc comprising 10 parallel capillary channels [111]. Using several of these discs in parallel enables scaling laboratory syntheses up to a small production scale. Although it is difficult to find the use of microreactors alone in flow analytical systems, microfluidics holds a solid position in flow analysis, which is shown later on in this paper. 

Yet another trend in this field is the use of monolithic reactors made of different types of macroporous materials such as polymers, polymer/glass, silica, or zeolites in flow synthetic systems [112]. As compared to batch or packed-bed reactors, they provide higher yields and improved selectivity, and, in many cases, they exhibit improved mechanical and thermal stability. Some other materials used for this purpose may also induce some additional catalytic properties. 

It is worth mentioning that, since the late 1990s, monolithic columns were employed to carry out high-performance chromatographic separations in both low-pressure FIA and SIA systems [115].

The common element which connects the instrumentation for flow analysis and flow synthesis is the design and construction of multistep manifolds, which are complete flow systems for the determination of the target analyte or for the production of target products from different substrates using different synthetic steps. Here, it is easy to find numerous similarities, although the symbols for their graphical presentation in the schemes published in the subject literature can slightly differ.

**Figure 1:**
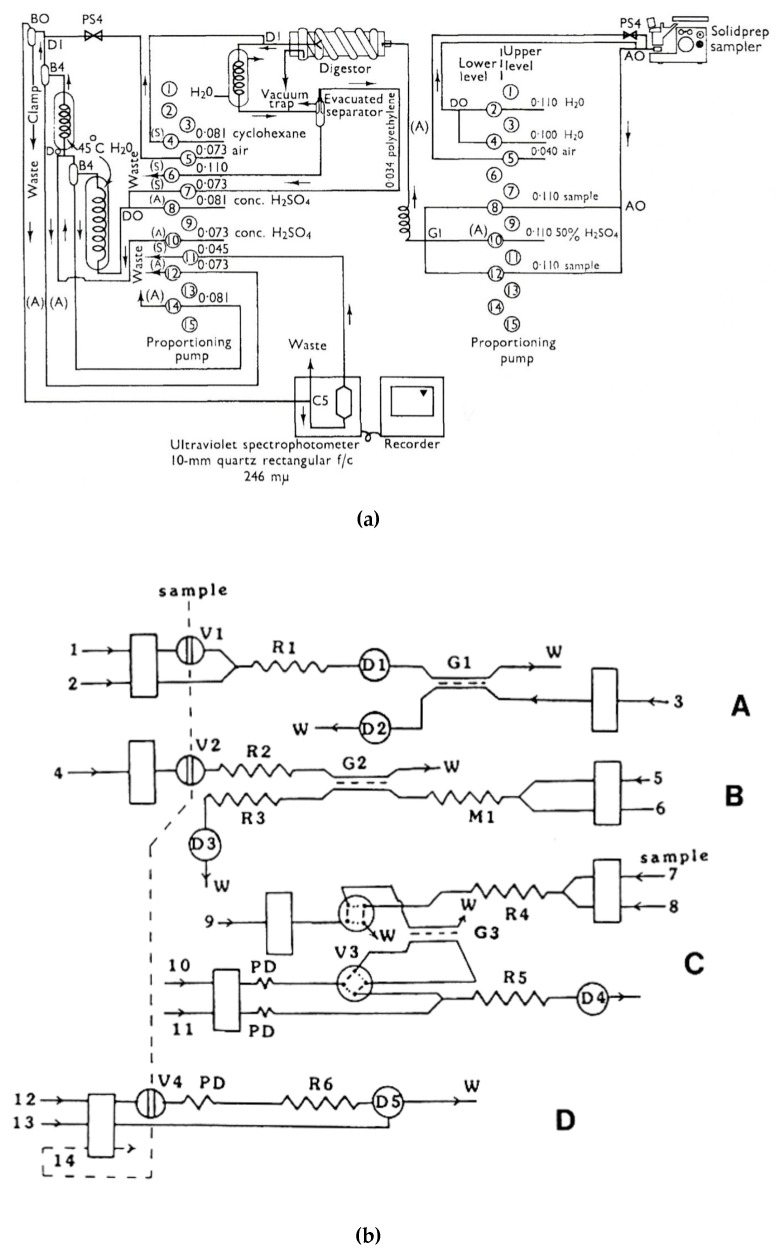
Schematic diagrams of multistep flow analytical systems: (**a**) air-segmented continu- ous-flow system for the determination of biphenyl pesticide in citrus fruit rind involving in-line distillation and ultraviolet (UV) detection [113]; (**b**) flow-injection system for simultaneous photometric determination of sulfide, polysulfide, sulfite, thiosulfate, and sulfate [114]. A—manifold for determination of sulfide and polysulfide; B—manifold for determination of sulfite; C—manifold for determination of thiosulfate; D—manifold for determination of sulfite.

Expanded manifolds for analytical determinations are constructed when multistep on-line operations of a sample treatment are necessary, as well as when analyte derivatization is necessary for a particular detection. The determination of the residues of pesticide known as biphenyl in the samples of citrus fruits, including on-line distillation among other operations, is a good example of a continuous-flow analytical procedure, reported in 1966 [113] (Figure 1a). The application of in-line distillation in continuous-flow chemical synthesis can be illustrated by the system with immobilized organic base catalysts developed for semi-continuous nitro alkene formation and Michael addition [116]. In this case, in-line distillation was used to remove the excess of nitromethane from the reaction mixture.

Yet another type of analysis requiring widely expanded manifolds with sample treatment steps is speciation analysis, where different compounds of the same element are simultaneously determined in the same sample. Some examples of such advanced flow analytical systems include those reported for the determination of four sulfur anions [114] (Figure 1b), chlorine-containing anions [117], or speciation of nitrogen compounds [118,119]. Some other expanded systems also comprise, for instance, an SIA system with atomic fluorescence detection employing cold vapor generation, constructed for the determination of labile and non-labile fractions of mercury in environmental solid samples [120], or flow systems with a sophisticated calibration procedure, allowing a reduction or elimination of the effects of interferences in the determination of calcium in dairy products with solenoid-type mini-pumps and flame atomic absorption spectrometry detection [121]. 

Multi-analyte flow systems are different kinds of determinations carried out under flow conditions in multiline systems. They were constructed in continuous-flow mode with stream segmentation (see, e.g., the system for determination of pharmaceuticals in tablets [122]), and they were further developed in the case of flow-injection methods [123,124]. In most of the above-mentioned FIA systems, the propulsion of fluids was carried out mainly with peristaltic pumps; however, syringe pumps are also quite widely employed for this purpose (see, e.g., multi-syringe systems [118,120,125]). They are especially indispensable in SIA systems including those with LOV and renewable sorbent beds where different directions of flow are commonly used. One more alternative, which was frequently used in recent years, involves solenoid minipumps and valves, especially advantageous for the design of fully computer-controlled flow analytical systems [121]. 

**Figure 2:**
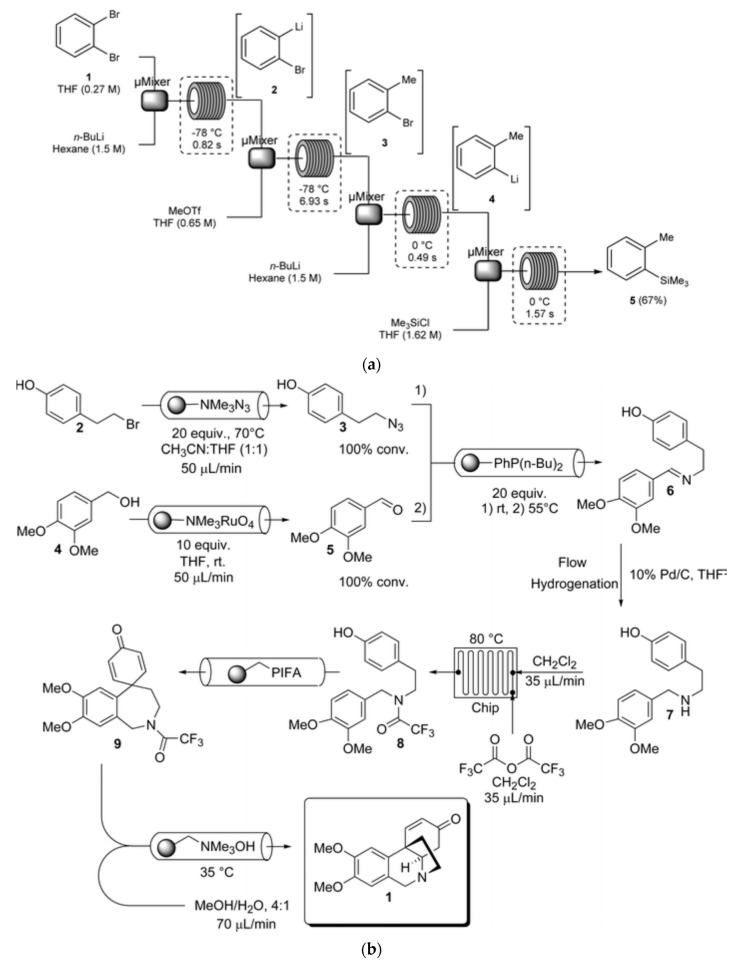

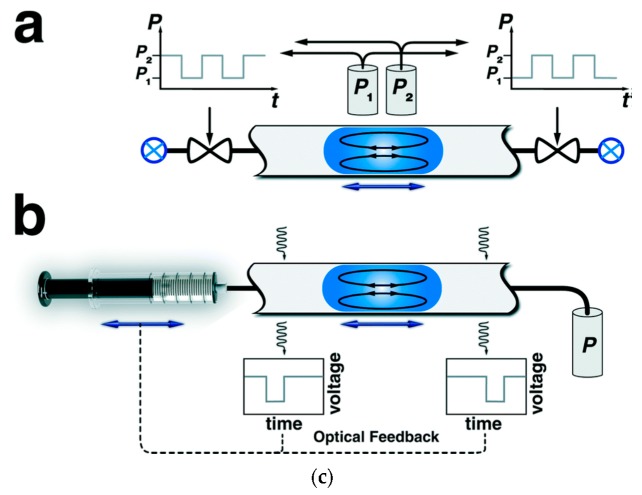
Schematic diagrams of two example multistep systems (**a**,**b**) and an oscillatory system (**c**) developed for flow synthesis: (**a**) a flow system with a so-called telescoping reaction sequence which involves consecutive reactions of products formed in the previous step via addition of new reagents or catalysts to the reactor [127] (adapted from [128]); in the system composed of four micro-mixers and four micro-reactors, sequential reactions of *o*-dibromobenzene with two electrophiles were carried out; (**b**) a flow system with various packed columns containing immobilized reagents, catalysts, and scavengers developed for the synthesis of the alkaloid natural product (±)-oxo- maritidine [7]; (**c**) basic types of oscillatory multiphase flow systems driven by valves (a) and a syringe pump (b) [134].

Multistep syntheses are common practice in organic laboratories. One type of widely used procedure in flow synthesis is the so-called telescoped reaction sequence, which involves consecutive reactions of products formed in the previous step via an addition of new reagents or catalysts to the reactor [126]. Piston pumps, commonly used in HPLC instrumentation, are usually used as a pumping device in such systems, and an example of such a configuration, developed for the bromine–lithium exchange reaction with *o*-bromotoluene [127], is shown in Figure 2a. All the reactions taking place here are very fast and exothermic; hence, the heat transfer is a critical issue. This is the case where, due to the high surface-area-to-volume ratio, conducting the reactions in microreactors is especially advantageous. Numerous multistep flow synthesis systems involve various physico-chemical processes carried out in different flow-through modules assembled in the whole set-up. Many of them can be found in comprehensive reviews [128,129,130,131]. A pioneering contribution to the development of such systems is usually attributed to Ley’s research group from the University of Cambridge (UK), including their work on the synthesis of peptides [132] and the multistep flow system developed for the first continuous synthesis of an alkaloid natural product called oxomaritine [7]. The latter study consisted of seven steps involving the use of packed columns with immobilized reagents, catalysts, and scavengers. A scheme of all reactions carried out in that particular flow synthesis is shown in Figure 2b. The possibility of creating such systems was well illustrated in a recent paper on reconfigurable systems [133]. The analogy to the above-mentioned works on analytical flow systems with various steps of sample processing or for multicomponent determinations seems to be evident.

The segmentation of the carrier stream in pioneering Skeggs’s continuous-flow analysis set-ups [12,13], together with those constructed and reported over the next two decades, served as a basic tool for the reduction of dispersion of a sample zone in the flowing stream. The process required the effective removal of air bubbles prior to the photometric detection cell; however, later on, debubbling was replaced by electronic controlling of photometric measurements without removing air bubbles. Such flow systems were usually constructed as multiline manifolds (Figure 1a); however, in a Technicon Chem-1 advanced commercial analyzer, segmentation was also used in a single-line system without debubbling [135]. This was performed in such a way that air bubbles were introduced only between each sample and the flowing flush solution [136], or into a so-called mono-segmented system where the sample was introduced between two air bubbles [137]. This also includes the set-ups in segmental flow-injection analysis [138], where the flow sample zone is surrounded from both sides by air bubbles to obtain the same effect of controlling dispersion along the tubing. Among some more recent examples of utilizing flow segmentation in analytical systems, one can find a microfluidic system designed for detecting bacteria and determining their susceptibility to antibiotics [139]. Plugs with aqueous solutions of different drugs are separated by fluorinated carrier fluid and mixed with a stream of examined bacterial solution and staining reagent stream. A degree of interaction was observed after appropriate incubation in a stopped-flow mode. 

A similar idea to maintain a discrete entity within plugs to carry out partial reactions is employed in flow synthesis systems. If the aqueous segments of reagents with the catalyst in fluorinated fluid in a Teflon microreactor are separated by hydrogen bubbles, this provides a successful set-up for carrying out a regioselective hydrogenation reaction [140]. Such a procedure, described as a plug-flow approach, enables carrying out chemical reactions on a μL–mL scale. Moreover, in the segments of different organic solvents transported in a perfluorinated carrier solvent, one can carry out the optimization of reaction conditions that can be then transformed into a large-scale batch reactor [141]. The efficiency of catalytic oxidation of methanol was examined in a segmented flow system with zones of different catalysts in a perfluorinated carrier, where, under high pressure, the mixture of substrate and oxygen was introduced through the Teflon membrane [142]. It can be concluded that the commercial success of clinical analyzers with segmented flow between 1960 and 1980 found a kind of continuation/replication in the process of designing segmented flow synthesizers to accelerate the generation of compounds in pharmaceutical discovery [143].

A particular mode of exploiting the stream segmentation in flow systems is also the so-called oscillatory flow strategy, where, as a result of the periodic reversal of a flow direction, a droplet of the reaction mixture in an immiscible inert carrier oscillates within the tube with, for instance, in situ UV monitoring [134], in either valve-based or syringe pump-based flow set-ups (Figure 2c). Such oscillatory multiphase systems can be used both for screening and for conducting chemical processes with a long processing time, and they are also suitable for carrying out kinetic investi- gations.

The basic part of each analytical or synthetic flow system is a fluid propulsion device. Since the creation of the first analytical systems in the 1950s, the use of peristaltic and syringe pumps predominated over the use of gas-pressurized reservoirs, gravity flow, or piezoelectric micropumps. A different alternative initiated in the early 1900s was the use of electroosmotic flow (EOF) [144]. However, it has its limitations due to the fact that the direction of EOF depends on the composition of the solution. On the other hand, the line with EOF can be hydrostatically coupled to propel other solutions. The applications of this concept in micro-flow-injection analysis systems were reviewed [145]. Then, some years later, this concept was also adopted for synthetic purposes within microreactors, and these approaches were also reviewed [146]. It was demonstrated that, for instance, EOF can be employed in systems with a continuous-flow reactor for solid-supported synthesis [45]. The solid-supported catalyst bed is placed in the microreactor where EOF is generated, and one of the reagents is immobilized on functionalized silica gel. With EOF is used in the capillaries, the volumes of samples/reagents are limited to nL–μL volumes; however, in the case of tubes packed, e.g., with solid catalysts, much larger diameters of tubes can also be employed without back-pressure, which allows building such flow systems up to a semi-preparative scale [147].

Yet another kind of analytical and synthetic flow system which functions as a result of the sequence of several operations carried out under flow conditions is the configuration including one operation conducted in a non-flow discrete mode. These systems are described as flow-batch systems and their development was initiated by describing an automated micro-batch analyzer with the sample loaded into an injection loop and blowing into the reaction chamber using compressed gas [148]. This was developed for various determinations using different detection techniques, followed some years later by the creation of flow-batch titrators for photometric titrations [149], as well as a spectrophotometric flow-batch system for the determination of aluminum in plant tissues [150]. In such systems, for instance, a series of standard solutions can be prepared in a discrete mixing vessel and introduced into the flow measuring system, which was reported in the set-up designed for trace determination of Mn with electrothermal atomic absorption spectroscopy (AAS) detection [151]. The flow system can be stopped with a non-flow detector, operating also as a mixing vessel, a phase separator, and a detector, which was reported for the set-up designed for the photometric determination of Fe(III) in oils [152]. One of the most recent reports dealt with the creation of a flow-batch system for the speciation of Cr(III) and Cr(VI) with chemiluminescence detection, based on the role of Cr(III) as a catalyst in the reaction of luminol with hydrogen peroxide, and Cr(VI) as an oxidant in the luminol reaction using chemically modified carbon nanotubes in the separation process [153].

Such a method of creating flow-batch systems is even more frequently employed in designing flow systems for organic syntheses, which was very well illustrated by a recent comprehensive review on the development of flow synthetic methods for the pharmaceutical industry [154]. In numerous cases, a 2–3-step telescoping process is ended in a batch reactor with a quenching step. This mostly concerns the works published in recent years, including, for instance, the preparation of boronic acid 6 [155], the generation of dichloromethyllithium [156], or the running of tube-in-tube reactions with diazo-methane in a batch reactor [157], among many others. As a particular achievement in this area, the development of an automated platform integrating batch and flow reactions should be pointed out [158]. This was based on the modularity of the flow process, where, in a more favorable situation, the flow module can be replaced by a batch reactor. Based on the complex synthesis of the precursor of an anticancer drug candidate, the creation of three phases of the whole synthesis procedure, incorporating batch and flow steps configured in a telescoped manner, took place. The whole system was computer-monitored/controlled and cloud-based with the server and the server–equipment interaction occurring via the internet. The selected citations from the literature indicate the development of very similar solutions in the design of flow systems for both analytical and synthetic purposes.

**Figure 3:**
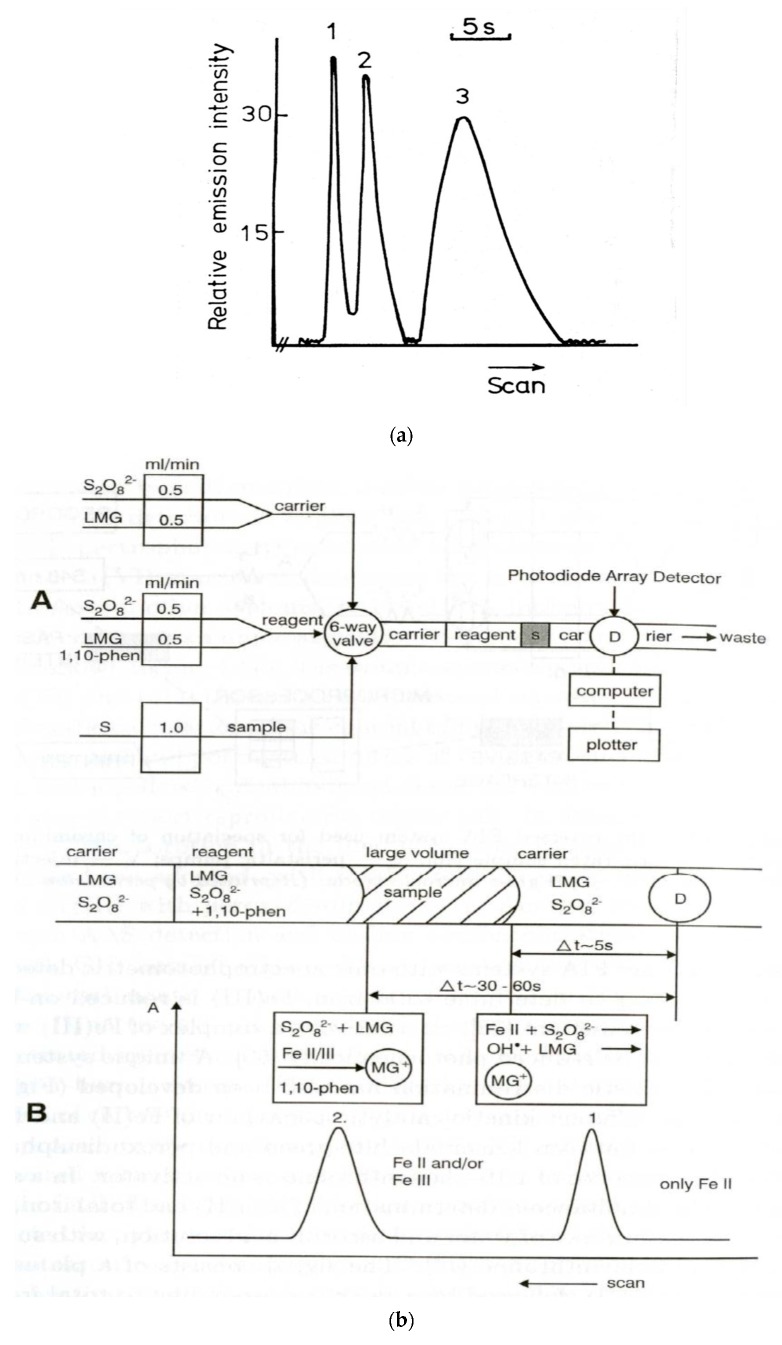

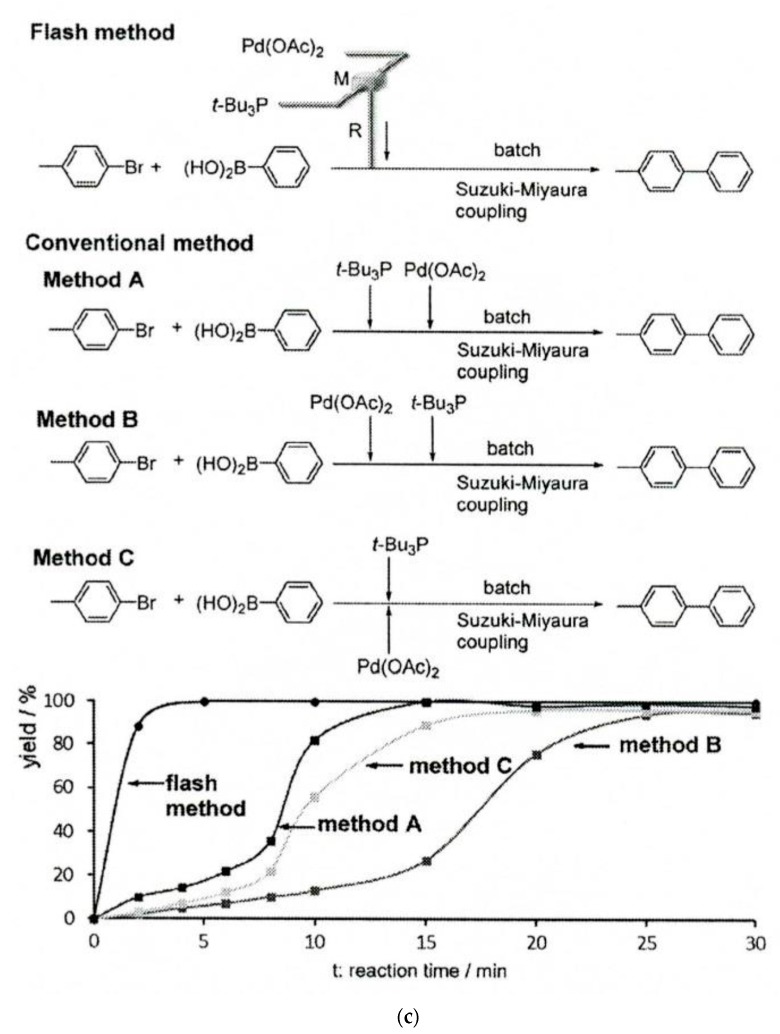
Examples of the kinetic effects for speciation analysis in flow-injection analysis (FIA) systems (**a**,**b**) and in continuous-flow synthesis (**c**): (**a**) flow-injection signal recorded form a single injection of mixture of sulfide, sulfite, and sulfate into an FIA system with a molecular emission cavity detector [159]; (**b**) manifold of the FIA system (A) and schematic diagram of the sample/ reagent sequencing (B) in the speciation of iron(II) and iron(III), utilizing kinetic discrimination of analytes in the reaction between leucomalachite green (LMG) and persulfate with 1,10-phenan- throline as an activator [162]; (**c**) optimization of continuous-flow synthesis procedure for Suzuki–Miyaura coupling of *p*-bromo-toluene and phenylboronic acid in the presence of KOH catalyzed by [Pd(OAc)_2_]-*t*-Bu_3_P [171].

A common feature of measurements and operations under flow conditions is certainly the possibility of both monitoring and controlling very fast chemical reactions. As already shown, this is the basis for using flow conditions in fundamental kinetic studies. In the very early years of the development of injection methods in flow analysis, very fast kinetics of the reaction of sulfur species in a molecular emission cavity detector was utilized for the speciation of sulfur anions [159] (Figure 3a). The differences in the kinetics of either the formation or the dissociation of complexes in a solution were utilized at the same time for multicomponent FIA determinations. This can be illustrated by the simultaneous FIA determination of Ca and Mg based on different reaction rates of the dissociation of cryptand complexes [160] or of Co and Ni based on different reaction rates of the formation of citrate complexes [161]. In the FIA system shown in Figure 3b, the speciation analysis of Fe(II) and Fe(III) was based on the reaction between leucomalachite green and persulfate with 1,10-phenanthroline as an activator [162], thanks to the different catalytic effect of these analytes. Among the fast processes utilized in flow analysis, numerous applications in flow-injection analysis were reported for chemiluminescence detection. It was demonstrated that, for instance, in the case of chemiluminescence resulting from the reaction between urea and hypobromite in an alkaline medium, the maximum emission occurred 2 ms after mixing the reagents, and it was completely extinguished after 400 ms [163]. This can be applied in analytical determination using a double concentric tube mixer, located directly in front of the photomultiplier tube. Such instrumentation was used also much earlier, e.g., to trace the chemiluminescence determination of Co based on the catalytic oxidation of luminol by hydrogen peroxide or sulfide, based on the fluorescein-sensitized oxidation of sulfide by sodium hypochlorite [164]. Among the organic analytes, a fast chemilumine- scence response was utilized, e.g., in the FIA determination of antihypertensive compound known as dihydralazine sulfate, based on the strong enhancement of luminescence produced by the analyte in the reaction of luminol with diperiodatocuprate [165]. It can also be pointed out that the differences in the rate of the emission of chemiluminescence can be utilized for multicomponent flow determinations, which was observed in the determination of two opiate narcotics, namely, morphine and naloxone, based on their oxidation by permanganate [166].

In the much further development of flow synthesis procedures, fast reactions are very widely employed enabling much faster procedures than in conventional batch systems. A convincing example can be, e.g., total synthesis of the fluoroquinoline antibiotic known as ciprofloxacin [167]. This was described as the longest linear sequence of reactions telescoped under flow conditions, which involves six chemical reactions carried out in five flow reactors with 9 min of total residence time. The corresponding patented batch syntheses take up between 24 and >100 h. In yet another example, the synthesis of carboxylic acids via a fast lithiation–carboxylation sequence at room temperature in continuous flow took less than 5 s [168], although a reaction time of only 32 ns was also reported [169]. These are time intervals exploited in fast chemiluminescence detections for analytical purposes. 

The field of chemical syntheses involving extremely fast reactions conducted in a highly controlled manner is called “flash chemistry” [170]. Conducting such reactions under flow conditions is especially advantageous and, in some cases, virtually impossible under batch conditions [171]. Only in a continuous-flow system can the reaction time be controlled by the residence time. The formation of unstable intermediates can be suppressed by the time-controlled addition of a quenching reagent. 

The flow synthesis approach with strict control over the residence time serves as a powerful method for protecting group-free syntheses. The illustration of this whole flash chemistry concept in a flow system can be the use of a highly reactive short-lived catalyst before its decomposition. Figure 3c shows the use of a Pt(OAc)_2_ catalyst in the Suzuki–Miyaura coupling of *p*-bromotoluene and phenylboronic acid, while conducting it in a flow system with 0.65 s of residence time essentially improved the process, as compared to various configurations of the batch procedure. Numerous very fast reactions within flash chemistry are strongly exothermic processes, and conducting them in flow systems is especially favorable due to the excellent heat transfer in the flow reactors, as compared to the batch mode. This can be additionally facilitated by carrying them out under cryogenic conditions. In continuous-flow systems, this can be achieved using a dedicated cryogenic flow reactor operating on compact refrigerator technology [172]. This type of treatment under flowing conditions is virtually not used in analytical flow systems.

### 3.2. On-Line Processes Supporting Flow Analysis and Flow Synthesis

The possibility of carrying out various physico-chemical processes on-line in a mechanized way, which provides reliable results of determinations and shortens the analysis time, is a special advantage of such a mode of conducting analytical determination since the creation and the development of pioneering constructions of flow analytical systems. Already in the first developed systems for the determination of different analytes in blood samples, this on-line treatment included the mixing of solutions, dialysis in a membrane module, and time-controlled incubation [12,13]. The subsequent decades of progress in this area resulted in the development of modules for carrying out many other processes including multiphase separation operations, thermal treatment, chemical and electrochemical on-line processing, and irradiation at different wavelengths. In analytical systems, these processes are usually performed in additional dedicated flow-through modules which are located between the sample introduction device and the detector. In flow systems for syntheses, there are various sequences of flow-through reactors or columns with immobilized reagents, scavengers, or solid sorbents that enable catch-and-release steps. This is the reason why there are some differences in the used terms; however, more important in this case is the role that a particular operation plays in the whole assembled system, i.e., an analytical or a synthetic one. It also has to be admitted here that some of these operations were selected in this presentation only for the comparison of analytical and synthetic flow systems.

**Figure 4:**
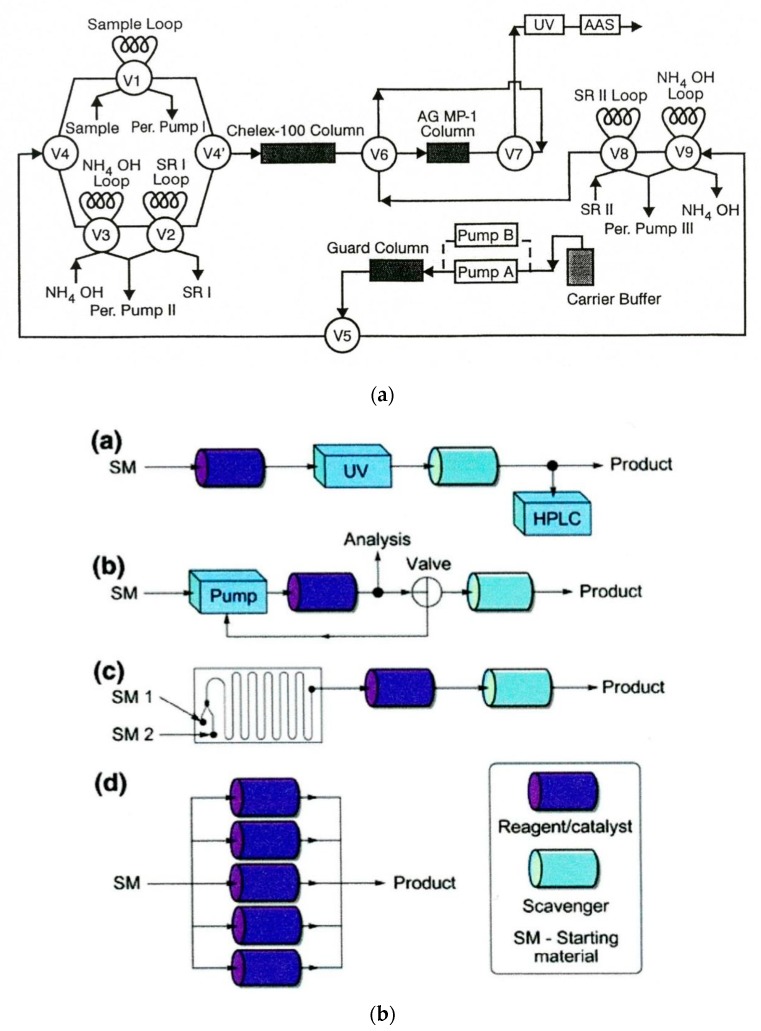
Examples of the application of solid sorbents in flow analysis (**a**) and flow synthesis (**b**) systems: (**a**) schematic diagram of flow-injection system with flame atomic absorption spectrometry detection for speciation of Cd, Cu, and Zn with the use of Chelex-100 chelating resin and anion-exchange AG MP-1 resin [184]; (**b**) schematic presentation of the main concepts of the arrangement of solid-supported reagents for continuous-flow synthesis [185]. a—linear configuration of series of flow-through column reactors with in-line and on-line analytical monitoring; b—recirculating set-up for processes requiring a longer reaction time; c—set-up combining streams and a flow-through reactor; d—parallel set-up of reactors for producing a larger amount of products.

The applications of solid sorbents in both flow analysis and flow synthesis were reported in both types of systems since the early 1980s. In analytical systems, they are used for the separation of analyte(s) from the sample matrix, along with sample clean-up, the preconcentration of trace analytes, and the immobilization of reagents, catalysts, and biocatalysts. Moreover, they are employed mostly as beds of beads packed in columns of different dimensions. In one of the earliest applications, bromide was preconcentrated on an ion-exchange resin in a segmented flow system [173], whereas ammonium ions were preconcentrated on a cationite in the FIA system [174], while a complexing sorbent was used on-line for the preconcentration of trace metal ions prior to their detection with flame AAS [175]. A very early example of using solid sorbents as an immobilizing reagent in analytical flow systems was the application of a cation-exchange resin in the silver form, from which silver ions could be released to remove chloride from the samples to be analyzed [176]. In addition to packed columns, solid sorbents can also be used in analytical flow systems, where they are attached to the internal walls of open tubular reactors [177]. This was also practiced for the immobilization of biocatalysts on the walls of a Teflon tube [178]. Solid sorbents can also be employed in the form of a suspension of magnetic beads with appropriate chemical functiona- lization [179]; they can be placed onto the tip of an automatic injection syringe [180] or as a renewable bed in the mini-columns of LOV valves, enabling the automated exchange of the sorbent bed [181]. The latter mode is especially convenient in the case of using rather instable solid sorbents such as, e.g., extraction resins widely used in determinations of radionuclides [182]. This commonly employed operation was the subject of numerous review papers and even a book [183]. A sample FIA system employing several sorbent columns for on-line solid-phase extraction in the speciation of Cd, Cu, and Zn is shown in Figure 4a [184].

The role of solid-supported reagents and scavengers in modern organic synthesis, with special emphasis on the generation of combinatorial libraries, was widely discussed in at least two reviews [186,187]. The main concept of the arrangement of solid-supported reagents for continuous-flow synthesis is presented in Figure 4b [185]. This can be a linear sequence of reactors, as described for the flow synthesis of yne-ones [188], a recirculation set-up for transformations which need a longer reaction time than the systems with microfluidics and sorbent-packed columns, or parallel set-up of reactors, for instance, for scaling up the production of a target compound. One example of particularly expanded systems with solid-state supported reagents was the above-mentioned first flow system reported for the total synthesis of a natural alkaloid [7] or a system for the synthesis of γ-aminobutyric acid (GABAA) agonists [189]. Some other examples were also reviewed [129].

Flow analytical systems were designed with the introduction of gases into the carried stream, based on the diffusion of selected components through suitable membranes, since the late 1970s. Usually, the goal of such a procedure is the separation of a given volatile analyte from the sample matrix to the carrier solution. For instance, gas diffusion through a membrane was employed in segmented continuous-flow systems for the determination of CO_2_ [190] and ammonia [191] in blood serum. In the continuous-flow determination of free chlorine in waters, the measuring flow system was equipped in a gas separator made of an internal porous Teflon tube and external non-porous Teflon [192]. A similar device developed later on for flow synthesis systems was called a “tube-in-tube reactor” [50]. In modern analytical flow systems, sandwich-type gas diffusion units are frequently used [193]. In flow synthetic systems, hydrogen was introduced to conduct hydrogenation [47], CO_2_ was introduced to carry out carboxylation [50], and CO was introduced to perform carbonylation reactions [194]. One can also find some examples of introducing gaseous chlorine, fluorine, oxygen, or ozone. In all these cases, the use of gases instead of various gas surrogates (e.g., aldehydes or metal carbonyls instead of CO) is advantageous from the point of view of product purification, although some limitations of that procedure may occur due to the poor solubility of reactive gases in various solvents [195]. Apart from the above-mentioned diffusion-based gas introduction into an appropriate membrane flow module, another employed possibility is the segmentation of the flowing liquid stream with the given gas or gas introduction in the form of an annular flow, where the liquid flows along the channel walls while the gas flows through the center. The latter mode was successfully used in a micro-fluidic system for conducting gas–liquid–solid hydrogenation [47]. Numerous applications of flow syntheses involving gaseous reactants can be found in many review papers [154,195].

Another unit operation conducted since the early developments of continuous-flow analytical systems with stream segmentation is distillation. This was reported, for instance, in some very early work on the photometric determination of alcohol and acids in beer samples [196] and in the spectrophotometric determination of total and free sulfur dioxide in wine [197]. One can find some other descriptions of such systems, for example, in the American Society for Testing and Materials (ASTM) standards. It seems that on-line distillation in flow synthetic systems may be helpful in removing volatile by-products or in separating the reagents or products from the unwanted materials. Recently, there were certain attempts to use flow distillation with the Hickman distillation apparatus [198] or so-called spinning-band columns [199], but such processes are not employed in the flow syntheses of pharmaceuticals [154].

In the course of multistep syntheses in continuous-flow systems, there may emerge the need for exchanging a solvent for different reasons. This may result from the necessity of maintaining on-line chromatographic control with the use of a particular mobile phase or a change of the reactor from a chemical to electrochemical one [200,201,202]. Certain flow modules to conduct such an operation were already designed in the early years of the development of flow analytical systems, for instance, as a moving belt interface for the hyphenation of a flow system with HPLC [203] or an ETD module (evaporation to dryness) [204]. Much later, yet another evaporating/condensing system was designed for the same purpose, i.e., switching the solvents in flow synthetic systems [205]. In a glass evaporator with a capillary sprayer, the concentric flow of gas assists the formation of fine spray of a solvent that rapidly evaporates. Then, the concentrated liquid is drawn out, while solvent vapor and carrier gas are directed to the condenser.

Both in analytical laboratories and in organic syntheses, microwave irradiation is used for heating samples or reaction mixtures for several decades now, which is actually also reflected in the construction of flow-through reactors for analytical and synthetic purposes. In analytical systems, microwave heating is applied to digest biological samples since the 1970s, shortening the analysis time from several hours to a few minutes [206]. In some pioneering on-line applications, it was used for the determination of Cu, Fe, and Zn in blood with atomic absorption spectrometry detection [207]. Another early example was the FIA system for the determination of chemical oxygen demand [208] or, more recently, the FIA system for the determination of total organic carbon with atomic emission spectrometry detection [209]. In the majority of syntheses under flow conditions requiring elevated temperatures reported, for instance, for the preparation of pharmaceuticals [154], mostly conventional heat sources like hot plates or oil baths are still used, although they are being more and more commonly replaced by microwave irradiation [41,210,211,212] or inductive heating [213]. Among different constructions of flow devices used for microwave irradiation in flow synthetic systems, one can find a coating of the flow reactor with a gold film for more efficient absorption of radiation [214], or capillary-based microwave reactor coated capillaries with a thin film of palladium [215]. In yet another approach, a U-shaped glass tube in a microwave-assisted system was filled with PdEnCat^TM^ beads, but it had to be followed by an additional column to remove residual palladium [216]. It seems that some of these designs can also be successfully employed in analytical flow systems. A low-cost microwave unit can also be manufactured from a fluorinated polymer tube and used with commercial microwave ovens [217].

Several interesting approaches were also reported in the application of inductive heating in flow synthetic systems. They include a flow-through microreactor with magnetic nanoparticles [218] and metal oxides such as CrO_2_ or NiO_2_ as bed material [219]. It was also demonstrated that a copper wire can be heated by induction and may simultaneously serve as a catalyst in the cycloaddition reaction [220]. 

**Figure 5:**
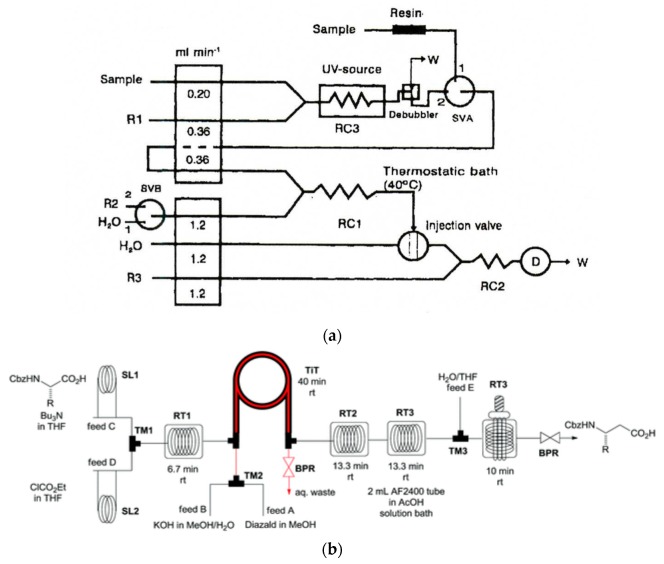
Examples of application of flow-through UV photoreactors in analytical (**a**) and synthetic (**b**) flow systems: (**a**) schematic diagram of flow-injection system with spectrophotometric detection for speciation of nitrogen in wastewater UV oxidation of organic species, nitrite, and ammonium into nitrate [118]; R1—persulfate alkaline solution; R2—reducing solution; R3—chromogenic reagent; resin—Amberlite XAD-7 sorbent; UV source—ultraviolet lamp; injection volume—90 μL; SVA, SVB—selection valves; RC1, RC2—reaction coils; RC3—photo-oxidation coil (3 m × 0.5 mm inner diameter (id)); W—waste; D—detector (540–420 nm); (**b**) schematic diagram of flow system for continuous-flow synthesis of β-amino acids from α-amino acids with photochemically induced Wolff rearrangement [226]; SL—injection valves; TM—T-mixers; RT—residence loops; TiT—tube-in-tube reactor; BPR—back-pressure reactors; PRT—photoreactor.

Similarly to several other attempts to create a flow system for chemical processing, UV irradiation steps were initially employed in flow analytical systems and, later on, widely introduced to flow synthesis. Among the early examples, one can find, for instance, UV irradiation in a segmented flow system for the determination of total cyanide using photometric detection [196]. As for flow-injection systems, one can indicate the determination of chloroorganic compounds using UV digestion and released chloride measurement using potentiometric detection with an ion- selective electrode [221], as well as measurements of dissolved organic carbon after oxidation in a UV reactor [222]. In an FIA system already mentioned for the speciation of nitrogen [118], nitrogen- containing compounds (organic compounds, nitrite, and ammonium ions) were oxidized in a flow-through UV reactor (Figure 5a). Among more recent publications, one can find, for instance, photochemically induced fluorescence, where, due to the UV irradiation of tigecycline (an analyte), a glycylcycline antibiotic, a fluorescent degradation product was produced, which was then determined in the FIA system [223].

Photochemical reactions were given increasing attention in recent years in the area of developing continuous-flow synthetic systems, as well illustrated by several reviews [85,154,211]. This also includes solar photoreactors [224]. The main advantages of using continuous-flow photochemistry in organic synthesis are as follows: short process time, high selectivity, the possi- bility of handling hazardous intermediates in a safe way, and straightforward scaling-up [85]. The advantage of photochemical reactors is the precise control of the energy input and the possibility of placing catalytically active species like TiO_2_ doped with Pt on the inner walls in microreactors, enabling the photocatalytic reactions to take place [225]. In the employed photoreactors, both UV and visible radiation are employed using mercury lamps as the source of radiation. Compact fluorescent lamps and white or blue light-emitting diodes (LEDs) are also used here. Numerous photoreactors of that kind are commercially available, and there were a number of developed synthetic routes which use them [85,154]. An example of a four-step synthesis process carried out with the use of a photoreactor was the production of β-amino acids from α-amino acids [226] (Figure 5b). A photocatalytic process involving the use of a photoreactor and irradiation from a white LED was reported for the aerobic oxidation of thiols to disulfides using eosin Y as a metal-free photocatalyst [227]. It can also be noted in this aspect of flow synthesis that numerous innovations achieved in the construction of photoreactors can also be successfully employed in analytical flow systems.

Similarly to photochemical processes, an increasing interest in flow synthesis was observed in recent years in the use of electrochemical processes, according to several recently published review papers [228,229,230]. Numerous applications of flow electrosynthesis for pharmaceuticals were also reviewed [154]. In this case, there are not as many similarities in the role and evolution of these processes between analytical and synthetic flow systems, as pointed out for many other aspects above. Basically, electrochemical methods in flow analysis are quite commonly used detection methods, both for inorganic and for organic analytes (in the latter case, mostly employing biosensors and on-line biochemical flow-through reactors). There are, however, some other cases of applying electrochemical processes in the area of flow analysis. This can be, for instance, the generation of very unstable reagents as U(III) [231] or Ag(II) [232] in flow systems, which is similar to flow electrochemistry in flow synthetic systems. In yet another application of an electrochemical reactor in an FIA system, the on-line electrochemical reduction of nitrate to nitrite was used in the spectrophotometric determination of nitrate [233]. Electrochemical dissolution can be used [234] for the flow-injection determination of the composition of metallic alloys. The generation of reagents by electrodialysis for a capillary-scale flow analytical system was also developed for this purpose [235].

The increasing number of applications of electrosynthetic processes was accompanied by a large variety of constructions of flow reactors [229] (especially flow microreactors [228]), a variety of employed electrode materials, and a variety of geometric configurations of reactors. Generally speaking, high surface-area-to-volume ratios of the cell and the proximity of electrodes in the flow cell are two factors that favor obtaining the highest yields. Among the most sophisticated designs of such reactors are the cells with an interdigitated electrode configuration [236] and packed-bed cells with a three-dimensional electrode [237]. Many flow electrochemical cells for flow synthesis are already commercially available, and such examples were shown in a review paper [154] together with some examples of applications involving oxidation, cyclization, dehalogenation, and C–C bond formation processes. One can expect that some electrode processes carried out for synthetic purposes with different flow-cell arrangements might also be also employed in the electroanalytical flow systems.

### 3.3. Microfluidics in Flow Analysis and Flow Synthesis

A significant technological breakthrough in both the above-discussed fields of flow chemistry was the instrumental down-scaling of flow systems to a microfluidics format. In the case of flow analysis, this was preceded in the early 1980s by a pioneering design of the first integrated miniaturized flow system called micro-conduits [238,239], while, in the case of flow synthetic systems, the beginnings can be attributed to the use of capillary microreactors. Moreover, in this case, the first attempts at developing microfluidic technology were associated with their analytical applications, as already mentioned in this paper. In general, it seems that, in the development of such systems for analytical purposes, in addition to composing the whole hydraulic part from capillary channels, a challenging task is to integrate as many operations (sample introducing, mixing, on-line pretreatment, detection) of the sample on the same chip as possible. In the case of chips for synthetic purposes, they are mostly designed as microreactors for carrying out operations such as a particular reaction or multiphase separation.

While the first design of microfluidic chips was designated for gas chromatography, their first applications in wet analysis took place in the middle of the 1990s, involving capillary electrophoretic determination of metal ions with laser-induced fluorescence detection [240] and the first designs of chips with integrated detectors [28,241]. In the early 2000s, apart from various microfluidic analytical systems, the first very successful applications of microfluidics for flow synthesis were reported at the same time [42,47,242]. Among the early analytical systems, a particularly original application was a single-channel glass microchip for fast screening (using flow-injection mode) or for detailed determination of nitroaromatic explosives (in capillary electrophoretic mode) or organophosphate nerve agents with amperometric detection [243]. A different mode of analysis was possible due to the use of different high-voltage polarization for the same chip, which allowed employing either flow-injection or electrophoretic measurements. 

Almost simultaneously, Kitamori’s group from the University of Tokyo reported the creation of a microfluidic chip for flow analysis with thermal lens microscopy detection, demonstrating numerous on-line micro-unit operations [244], as well as a microfluidic device for conducting gas–liquid–solid hydrogenation reactions [47].

New analytical applications of microfluidics included, for instance, the creation of a generic microfluidic system for immunoassays with electrochemical detection [245] and a continuous-flow microreactor for cyclical reactions to perform polymerase chain reactions, which can be used for DNA analysis [6]. On the other hand, two microfluidic systems were reported for the synthesis of radiolabeled compounds. One-step synthesis of ^11^C- or ^18^F-labeled carboxylic esters was demon- strated in a hydrodynamically driven microreactor [242], while, in yet another approach using an integrated microfluidic chip, a five-step ^18^F-radiolableled imaging probe for PET was synthesized [42].

The extremely intense development of analytical microfluidic systems brought a variety of designs which involved carrying out various sample treatment operations, as well as various types of detections. These include, for instance, the integration of preconcentration on a molecularly imprinted polymer with sensitive spectrophotometric detection [246] or the hyphenation of a microfluidic system for thermospray sample introduction with flame atomic emission spectroscopic detection [247]. The appropriate design of a single handheld electronic system may offer the possibility of using different configurations of microfluidic systems with different detectors [248], where point-of-care diagnostics seems to be a particularly important and attractive area of application [249].

As already pointed out, microfluidics and microsystems in general are regarded as the most promising devices to enhance the drug discovery processes [40], and, depending on the need for a particular sequence of processes, both microreactors and micro-separation units can be arranged in different combinations [49]. The greatest advantage of microfluidics is the possibility of conducting reactions which cannot be carried out in conventional glassware. This concerns, for instance, using extremely high temperature and pressure, using micro-amounts of substrates, working in sealed systems, or minimizing the contamination of water or oxygen [250]. This was widely illustrated by the vast literature of original research works, including numerous review papers [108,228,251,252,253,254,255].

The objective of both evolution and continuous searching in the construction of microfluidics for flow analysis and flow synthesis is looking for the most appropriate material for their fabrication. The selection of such building materials depends mostly on the target application of a device; however, it also depends on the cost of mass production and the flexibility in preparing the prototypes for research purposes. The capillary micro-structured reactors for flow syntheses are made of silicon–Pyrex, ceramics, stainless steel, or glass [108]. Microfluidic devices for analytical purposes are mainly produced from polydimethylsiloxane (PDMS) due to its low cost, robustness, and simple procedures of fabrication; however, among other polymers employed for their preparation, one can also find thermoset polyester, polyurethane methacrylate, and photocurable resins [256]. Low material consumption and using low-cost materials are the main advantages of the preparation of microfluidics for analytical applications on thin and flexible films [257]. Then, ultra-low-cost microfluidic devices can be produced using polyolefin shrink film with a digital craft cutter involving thermal bonding of the layers [258]. A so-called green alternative to the above-mentioned materials is the use of corn proteins when creating analytical microfluidic devices, where thin zein films with microfluidic chambers and channels are made and bonded to the glass slide with the use of standard lithographic techniques [259]. 

Since the pioneering work by the Whitesides group on 3D microfluidic devices produced by stacking layers of patterned paper [260], paper-based microfluidic devices gained quite high attention in the analytical chemistry community [261,262]. Able to operate without external pumps owing to strong capillary actions, they are especially appropriate for mass production, healthcare applications, and water analysis [263]. Examples of analytical applications include, for instance, the use for automated staining of malaria parasites prior to microscopic detection [264] and photometric determination of ammonia in freshwater with the device employing a micro-distillation chamber [265].

Teflon-patterned paper was employed in the flow-through synthesis of 100 peptides of 7–14 amino acids [266]. The obtained peptide arrays were used in a cell-based screening to identify the bioactive peptides. In another approach, a flexible cloth-based microfluidic device for analytical purposes was prepared by carving a designated pattern on wax-impregnated papers and attaching it to a cotton cloth via heat treatment [267].

Microfluidics is used for conducting chemical processes in separated droplets both for synthetic and analytical purposes, since the early 2000s. The advantages of such an approach were exploited for the above-discussed analytical and synthetic flow systems with the segmentation of flowing liquid with gas bubbles or immiscible liquids. Droplet-based reactors also operate well on a larger scale, which was demonstrated, e.g., in the reactions carried out in aqueous droplets with catalytically active interfaces to perform Suzuki–Miyaura coupling reactions using a fluorous- tagged palladium catalyst [268]. The main advantage of such a system is preventing fouling by isolating the reaction from the channel walls. 

**Figure 6:**
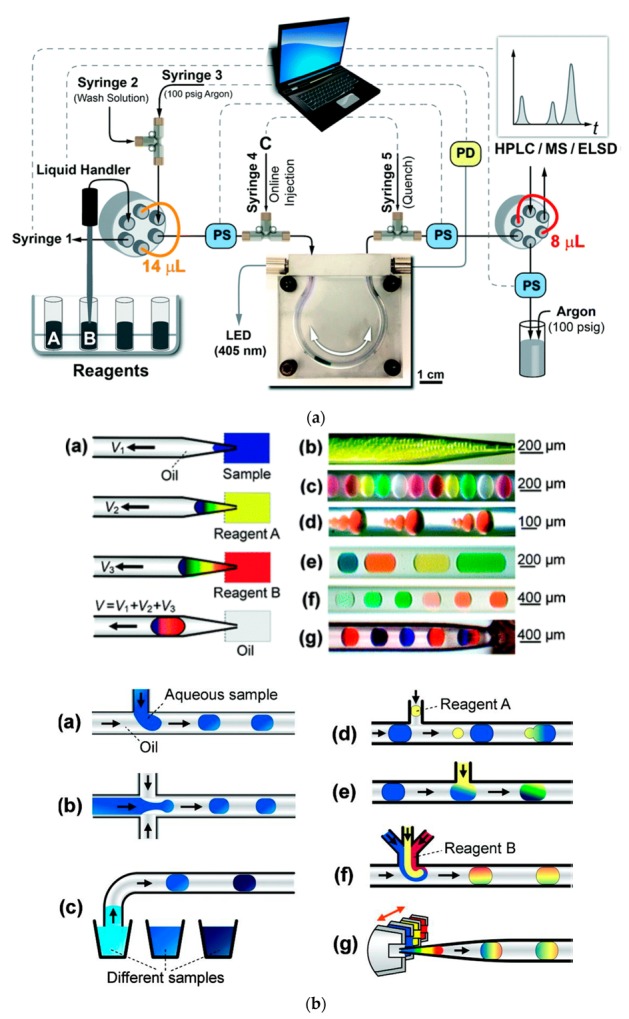
Examples of droplet-based flow systems developed for synthetic (**a**) [269] and analytical (**b**) [270] purposes: (**a**) schematic of the automated droplet-based medicinal chemistry platform [269]. PS—phase sensor; PD—photodetector. Dashed lines indicate personal computer (PC) commu- nication, gray lines correspond to the optical fibers for the light-emitting diode (LED) and the photodetector, and solid black lines correspond to the fluoropolymer tubing for the delivery and routing of liquid droplets; (**b**) principle and performance of DropLab [270]. (a) Schematic diagram of principle for assembling a three-component droplet using DropLab; (b) continuous generation of 20-pL droplets with a throughput of 4.5 s per droplet by sequentially aspirating a 20-pL fluorescence solution and 80 pL of oil at flow rates of 2 and 8 nL/min, respectively; (c) continuous generation of 1.6-nL droplets containing five different dyes; (d) droplets with different volumes of 20, 40, 160, and 1000 pL generated with 0.6 s of sampling time and increasing flow rates from 2 to 100 nL/min; (e) droplets with different volumes (2.5, 4.5, 5.5, and 8.0 nL) and different dyes; (f) series of 2.5-nL droplets with concentration gradient formed by diluting the green and red dye solutions with volume ratios of 1:19, 1:4, and 19:1 (dye/water); (g) 2.5-nL droplets formed by sequentially introducing red dye, water, and blue dye with different mixing ratios of 1.0:1.5:0, 0.5:1.5:0.5, and 0:1.5:1.0; illustration of various droplet generation modes used in droplet-based microfluidic systems based on T-junction (h), flow-focusing (i), and cartridge (j) techniques; various reagent mixing modes for droplets based on droplet fusion (k), post-mixing (l), and pre-mixing (m) techniques; (n) droplet assembling mode combining droplet generation with reagent mixing.

In another mode, controlled multistep synthesis can be carried out in a three-phase droplet reactor, where inert gas maintains uniform droplet spacing, which was shown in a five-stage quantum-dot synthesis [271]. An even more sophisticated system was invented for in-demand medicinal chemistry with the use of oscillating droplets for multistep synthesis allowing precise control of the reactor temperature and residence time for each step [269] (Figure 6a).

Even earlier than the above-mentioned works, the concept of droplet-based synthetic flow systems was employed in microfluidic formats, providing rapid mixing of reagents and no dispersion, with the suggestion of its application for both chemical analysis and synthesis [272]. In fact, the same research group employed such microfluidic droplet-based systems for the multistep synthesis of nanoparticles, which was performed on a millisecond time scale [273], as well as for the above-mentioned detection of bacteria and determination of their susceptibility to antibiotics [139]. The idea to miniaturize the instrumentation for carrying out polymerase chain reactions [274] is present since the middle of the 1990s, and a nanoliter-sized droplet-based microfluidic set-up was developed for this purpose [275]. With the amplification efficiency comparable to benchtop reactions, the total reaction time was reduced to one-half of that required for benchtop PCR instrumentation. One of the analytical droplet-based microfluidic systems reported in the literature was the automated microfluidic screening assay platform called DropLab. It was developed for various applications including enzyme inhibition assays, protein crystallization screening, and the identification of trace reducible carboxylates [270]. Numerous methods of droplet generation are shown in Figure 6b, together with different modes of the performance of a measuring system. The ionic liquid-based droplet microfluidic set-up for “on-drop” separations and colorimetric pH sensing, involving the creation of two-phase droplet structures in a continuous phase of silicon oil, is yet another example of a wise use of the above-discussed concept [276].

Here, so-called digital microfluidics is also worth mentioning. This concerns the manipulation of discrete volumes of liquids on a surface [277]. The concept of such a device was invented in the late 1990s, employing various mechanisms of the actuation of droplets on the surface such as electro-wetting, surface accounting waves, magnetically controlled droplet movement, dielectro- phoresis, or thermocapillary transport. There are numerous analytical applications of this device, for instance, for immunoassays of pathogens [278] in chemical synthesis, [279] or even in synthetic biology [280]. The developed processes with the movement of droplets and non-flow detection are considered to be discrete systems, not flow chemistry procedures; hence, they are not further discussed here.

Pressure-driven microfluidic chips are commonly manufactured using standard soft-litho- graphic techniques. These include, among others, prefabricated microfluidic assembly blocks that can be assembled to form a required microfluidic system for particular needs [281]. Since the middle of the 1990s, a new avenue in micro-constructive manufacturing was given by so-called 3D-printing, i.e., additive layer manufacturing. This was introduced in the early 2000s in the process of designing and fabrication of different instrumental parts and modules for flow chemistry, for both synthetic and analytical systems [282]. Additive manufacturing involves several basic techniques such as stereolithography, multi-jet modeling, selective laser melting, and fused deposition modeling to build a target object layer-by-layer. The main advantages of 3D-printing are its construction cost and production speed. It offers the production of extremely complex geometries; nevertheless, only a limited number of polymeric materials can be used with this technique, and some additional treatment, e.g., electron beam irradiation can be helpful in obtaining satisfactory mechanical properties of 3D-printed elements [283]. The examples of 3D-printed modules for flow synthesis include different flow-through reactors [51,284,285] and microfluidic chips for the synthesis of Ag and Au nanoparticles [286]. A 3D-printed microfluidic system was also produced for the generation of microdroplets, which can be used when creating functionalized microparticles [287].

There were numerous studies on the production of 3D-printed elements for flow analytical systems, such as flow-cells for chemiluminescence [288], as well as UV–Vis and fluorometric measu- rements [289]. Such devices incorporating disc-based solid-phase extraction were reported for the FIA speciation of iron using spectrophotometric detection [290], as well as fluorometric determination of Cd and Pb in waters using the LOV FIA system [291]. Many more examples can be found in a review on the analytical applications of 3D-printing devices [292].

The application of 3D-printed devices in flow systems combining synthetic and analytical functions seems to be particularly interesting from the point of view of the subject discussed in this paper. A 3D-printed reactor was used for the on-line preparation of Prussian blue nanoparticles in an FIA system with the amperometric detection of hydrogen peroxide. The nanoparticles were further employed for the modification of the working gold electrode, incorporated into a 3D-printed flow cell [293]. Then, in another approach, 3D-printed glass microfluidics was employed with the on-line mass spectrometry monitoring of linezolid synthesis [294].

Concluding this section of the present review, it should be pointed out that, in the field of the novel design and fabrication of microfluidic flow systems, new trends and developments are parallelly introduced and utilized in flow synthetic and analytical systems. In both areas, it is already well documented that these systems provide broad perspectives for many practical applications.

## 4. Toward the Automation of Chemical Flow Systems

The notions “to automate” or “automation” and their various derivatives are very loosely used when describing chemical instrumentation. The use of mechanical devices to replace, refine, extend, or supplement human effort in operating chemical instrumentation, including flow analytical and synthetic systems, does not mean automation of the instrument, which was clearly indicated already in 1994 in the International Union of Pure and Applied Chemistry (IUPAC) terminological recom- mendations [295]. Actually, the description covers only a different degree of the mechanization of instrumentation or experimental procedures. The word “automation” means introducing to such a procedure at least one operation which is entirely controlled and carried out by the feedback from the system (usually a computerized one) without any human intervention, which means self- monitoring or self-adjustment.

In the recent literature on flow synthesis, there were certain suggestions of how to diversify different flow synthesis systems, i.e., by differentiating between “automated” and “autonomous” flow synthesis systems [296]. As can be read, *automated* systems require human input to determine the boundaries of operating parameters, thresholds, and protocols, while *autonomous* flow synthesis systems can react to output parameters of the system such as, e.g., reaction yield or purity of the obtained products without human input. The employed control system should properly adjust the input parameters of the process. It seems that these two terms correspond exactly with the terms “mechanization” and “automation” in the above-mentioned IUPAC recommended terminology. This looks like another example of that invisible boundary between the decades of development of flow analysis and the re-discovery of both metrological and instrumental features for synthetic chemistry under flow conditions.

### 4.1. In-Line Analytical Monitoring in Flow Synthetic Systems

An application of real-time analytical monitoring of the progress of reactions carried out in flow synthesis systems is a crucial factor influencing the yield and the quality, i.e., the purity, of the fabricated product. Several decades of development of flow analysis and liquid chromatography methods resulted in the creation of a large number of flow-through spectrophotometric and electrochemical detectors, reported in the vast literature and commercially available from specialized manufacturers. Flow-through detectors for infrared spectroscopy are rarely used in flow analysis or LC, but they can be used in flow synthesis for the monitoring of selected compounds [297,298,299]. Raman spectroscopy analysis using a surface-enhanced Raman scatter (SERS) technique under flow conditions was also reported [300]. A number of examples were already presented in the literature on the application of NMR in flow analysis, e.g., in the determination of model drugs [301] or quantitative metabolome analysis of urine [302]. FIA systems can also be designed with mass spectrometry detection, for instance, for rapid determination of pesticides [303] or metabolome studies [304]. These randomly selected examples show the very broad experience gained in the use of the discussed sophisticated instrumental techniques in flow systems, which obviously can be adapted for real-time monitoring of the performance of flow synthesis systems.

One can find numerous examples of using analytical flow measurements for in-line monitoring among a large number of papers on flow synthesis. Most commonly used for this purpose are molecular spectroscopy techniques. For instance, in flow synthesis systems requiring minimal manual intervention, which were designed for the synthesis of a drug known as imatinib, a flow-through UV detector was used to determine when the reaction mixture exited the system in order to fractionate the reactor output for the off-line LC/MS analysis [201]. In the flow synthesis of fluorescent CdS nanoparticles in a microfluidic reactor, an in-line spectrometer to monitor the fluorescence spectra was employed [305], while, in a multi-stage flow synthesis of silica and two types of organic nanoparticles, in-line dynamic light scattering was used for real-time monitoring of the size of the obtained nanoparticles [306]. Numerous applications were reported for the use of infrared spectroscopy in real-time monitoring in flow synthesis systems, mostly using the so-called attenuated total reflection technique (ATR). Such a flow-through cell was attached, e.g., to the outlet of the electrochemical microflow reactor to monitor the on-line formation of the cationic intermediate [307]. An FTIR device with flow cells for ATR measurements using a gold-sealed diamond sensor (ReactIR) can be attached in-line at any part of the flow synthesis system to monitor the reagent consumption or product formation, as well as short-lived intermediates. It was employed, for instance, in the monitoring of fluorination and hydrogenation reactions, as well as the heterocycle saturation reaction for product monitoring; it was also used when screening reaction conditions, monitoring reactive intermediates in Curtius rearrangement, and monitoring hazardous wastes [308]. The last case was also separately presented showing the continuous monitoring of hazardous azide contaminants for the round-the-clock operation of a flow system in a fully automated fashion [309]. A multichannel Raman system equipped with ball-probe immersion optics was reported for monitoring each of the reagent lines and the product line in a flow system for the esterification of benzoic acid.

The hyphenation of flow synthesis with an appropriate analytical technique can be very helpful for quick optimization of the flow parameters and screening of the combinations of reagents. This was well illustrated by the so-called μSYNTAS system (microchip-based synthesis with total analysis system), which integrated the microreactor with a time-of-flight mass spectrometer via an electro-spray unit [310]. It was employed for the efficient continuous-flow generation of compound libraries on the microscale with real-time identification of the reaction components. In yet another example of applying such hyphenation, the microflow synthesis system was coupled to a miniaturized electrospray ionization (ESI) mass spectrometer to monitor reactive intermediates, screen starting materials, and optimize reaction parameters. This was employed for the generation of benzyne and its subsequent reaction with furan, showing the ability of such a hyphenated system to be adapted for safe operation due to the monitoring of hazardous intermediates and the generation of the desired target products [311].

An especially expanded flow synthesis platform, integrated in-line with a UPLC–MS set-up and made of commercially available components, was recently reported for both nanomole-scale reaction screening and micromole-scale synthesis [312]. It is characterized by enabling the preparation and analysis of up to 1500 reaction segments in a 24-h period. In a robotized synthesis–purification–sample-management platform, the preparative LC/MS system was integrated with the flow synthesis set-up for the generation of pharmaceutically active substances, where a 1000:1 split of a preparative system was directed toward another mass spectrometer for analysis [313]. Direct hyphenation of the HPLC set-up to a flow synthesis system can also be used for the same purpose, which was demonstrated, e.g., for a thermal isomerization reaction carried out in a flow reactor [314]. For the efficient generation of structure–activity relationship data, the flow synthesis system was implemented with a microfluidic biosensor chip employing fluorimetric detection [315]. It was developed for the preparation of the most active inhibitors of β-secretase (BACA1), considered as a key target in the research on Alzheimer’s disease.

### 4.2. Automated Flow Analytical and Synthetic Systems

In spite of the above-mentioned terminological recommendations [295], calling the flow analytical or flow synthesis systems automated is a misnomer. However, in the vast literature on flow chemistry in the fullest sense of the word, one can find several examples of really automated set-ups.

Titrations belong to the category of classic, yet still widely used, analytical quantitative procedures routinely employed in modern analytical chemistry, which are also adapted in flow systems. A very original concept which tells us how to carry out titrations is a so-called “binary search strategy”. It is based on a computer-controlled adjustment of the volumes of segments of a titrant and a titrand in order to reach stoichiometry, corresponding to the end point of titration. This is carried out in a computer-controlled system with automatic control and adjustment of the volume of segments using solenoid valves. Such systems were developed for acid–base titrations with photometric detection [316] and for the determination of copper complexation capacity, e.g., of milk, based on complexometric titration with chemiluminescence detection [317]. Two other examples of analytical automated flow systems concern the FIA set-ups with spectrophotometric detection developed for the speciation of Fe(III) and Fe(II) in water [318], as well as the simultaneous determination of thorium and uranium in environmental samples with the use of separation employing an extraction resin [319]. Different measuring procedures were programmed in these two both entirely computer-controlled measuring systems, and their selection for particular samples to be analyzed was made automatically based on a series of preliminary test measurements without human intervention. The main differences between the advanced programmed procedures are, e.g., the selection of an additional preconcentration step of analyte(s) or the dilution of the analyzed sample. 

Although, in a very recent paper [296], one can learn that “future advances may include the hyphenation of online reactor analytics that would allow a read-out of the reaction progress to alter the reaction parameters using feedback algorithms”, and that it can be achieved in the upcoming years, some examples of this approach can already be found in the literature on flow synthesis. For instance, in the designed microfluidic platform for multidimensional screening, chemical reactions were evaluated employing multiple variables [320]. UV on-line monitoring was utilized in such a system in a feedback loop to control both the selection and the injection of a reagent, setting temperature, and sample collection, while the analysis of the reaction screen was carried out using UPLC/MS in the samples collected into 96-well plates. Then, an infrared flow-through detector was employed in another multistep segmented flow processing system for the synthesis of pyrazoles to control the pump for the accurate addition of reagents in combination with a LabVIEW software application [321]. It was also mentioned that the output from the infrared cell can also be potentially used, e.g., to control a fraction collector in a fully automated process. 

The creation of a fully computerized autonomous flow chemistry platform for the studies of rhodium-catalyzed hydroformylation reactions was reported with the use of photodetectors, phase sensors, and mass flow controllers, but no information on the employed feedback loops was provided [322]. In some recently published papers, two extremely advanced and fully computerized systems for the flow synthesis of organic compounds were described for the synthesis of pharmaceuticals [52,312]. Both systems are reported as automated platforms, but no details on the use of the feedback loops for self-adjusting of any operations in their functioning are available.

### 4.3. Robotics, Digital Transmission, and Camera-Enabled Techniques in Flow Chemistry Instrumentation

**Figure 7:**
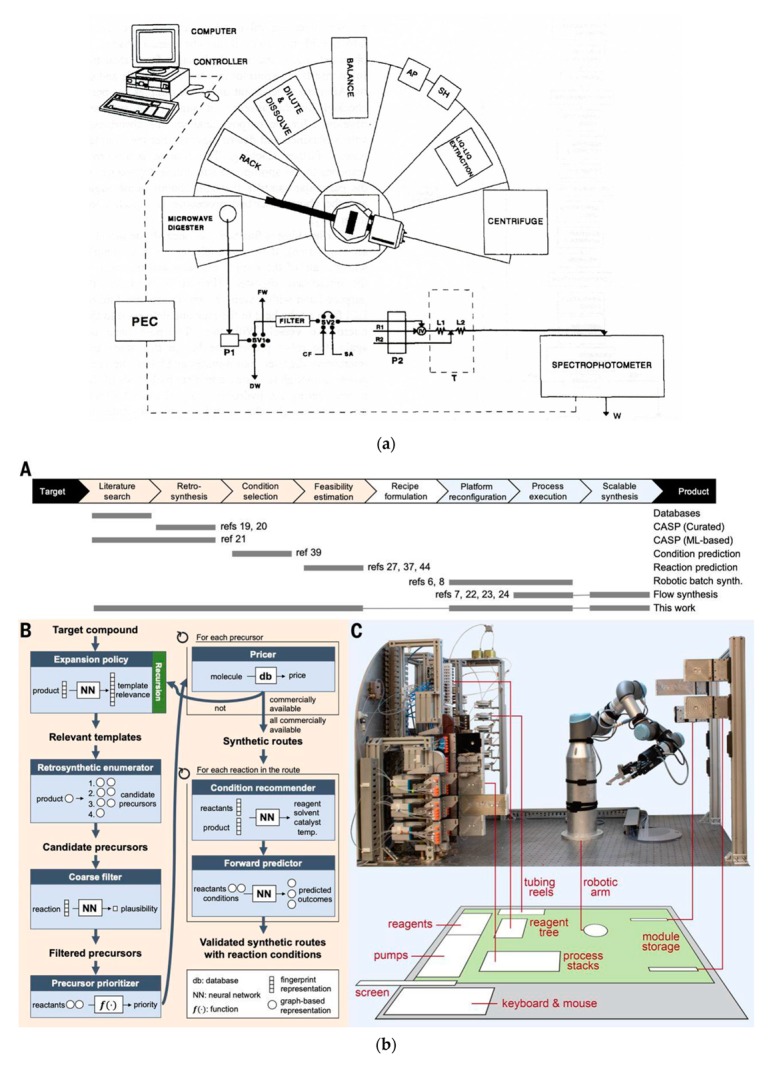
Examples of robotic systems developed for flow-injection analysis (**a**) [326] and flow synthesis (**b**) [52]: (**a**) FIA system developed for colorimetric determination of starch in food based on the determination of sugars using the neocuproine method; PEC—power and event controller; Ap—all-purpose hand; SH—syringe hand; P—peristaltic pump; FW—filter waste; DW—digester waste; CF—clean-up filter solution; SA—solution for assaying sugars; SV—switching valve; Rl—neocuproine channel; R2—NaOH channel; IV—injection valve; L—reactor; T—thermostat; W—waste; (**b**) robotic flow system for flow synthesis of organic compounds based on artificial intelligence planning; A—workflow for on-demand synthesis of a targeted organic compound; B—software modules combining cheminformatics and machine learning to design and validate synthetic pathways; C—photograph of the robotic flow chemistry platform, with a projected floorplan of the 6 × 4 ft working table.

A particular improvement in terms of the mechanization of complex, multi-modular instrumental systems, as well as their functioning in chemical laboratories, is the application of robots in their design. They offer significant technical enhancement in multistep procedures. Moreover, decision feedback loops can also be involved in their functioning, providing additional elements of automation to the operation of the whole instrumental set-up. A robot can be defined as a programmable, multifunctional machine which is capable of carrying out different operations using materials, parts, and specialized tools. In their second generation, they were equipped with numerous sensors, which make them automatic devices. As manipulators in the laboratory environment, they are designed in Cartesian, cylindrical, and spherical configurations. Their applications in chemical analytical laboratories for sample preparation were observed since the early 1980s [323]. In the same decade, their first applications for flow-injection analysis were reported for the preparation of oil samples prior to analysis using inductively coupled plasma (ICP) emission spectrometry [324]. A full description of a robotized sample preparation station was published in later works on the flow-injection photometric determinations of total polyphenols in olive oil [325] and starch determination in food products [326]. Figure 7a shows a scheme of a robotic station with a commercial, cylindrical-type robot called Zymate II Plus for sample preparation, involving sample weighting, microwave digesting, dissolving, and solvent extraction, as well as centrifugation [326]. The progress in the analytical applications of robots observed in next decades showed their essential advantages in the improvements achieved in small laboratories, in industrial research and development laboratories, and in the production of chemicals [327].

It seems obvious that the application of laboratory robots may also improve multistep and multi-instrumental systems for conducting organic syntheses in flow systems, especially when a given procedure requires manipulation with numerous reagents and different modules of the system. The discussed works were published in the most recent decade and most of them reported developments with the contribution of widely known pharmaceutical companies (Pfizer, GlaxoSmithKline, Abbvie). A Kawasaki six-axis robot was used for liquid handling in a flow system for the monophasic synthesis of drug-like compounds via a plug-flow approach already mentioned in this paper [141]. Using a nucleophilic aromatic substitution reaction and diazo transfer, it was demonstrated that the developed system for performing chemical reactions on a microliter scale can be scaled up to provide a much larger quantity of the required product by working with larger plugs. In another example of a robotized flow synthesis system, a Mitsubishi six-axis robot was also employed to hand off samples between different instruments involved in the whole process [313]. These stations included a segmented flow-chemistry reactor, a preparative LC/MS purification set-up, pumps for dilution, a labeling station, and a centrifugal evaporator. The process ended with MS and NMR analysis; the multistep reaction-time was only about 30 min due to the possibility of carrying out various parallel operations, which significantly shortened the overall production time for the library. A combinatorial synthesis robot incorporating a continuous hydrothermal reactor was developed in the area of direct solid-state chemistry and employed in the flow synthesis of Zn–Ce oxides as crystalline nano powders [328] and iron-doped lanthanum nickelates, which are difficult to prepare in a single step [329].

The most advanced robotic platform for the above-mentioned flow synthesis [52] was recently reported. It employs artificial intelligence planning and generalizes millions of known reactions, via which the prediction of a particular route for execution in a modular continuous-flow platform can be made (Figure 7b).

The whole constructed robotic platform consists of two towers containing eight universal process bays, connecting fluidic, electrical, and pneumatic lines using the UR3 Universal Robot^®^. The selected process modules are moved from the storage location and configured into a given manifold. The workflow for retrosynthetic planning firstly identifies the reactor for a given target compound; then, the generation of precursors and a proposal of the reaction conditions take place. In the developed instrumentation, ATR–FTIR was only occasionally employed in-line; however, in further modifications of that set-up, in-line HPLC and frit will be implemented for the optimization of the robotic platform by designing an analytic tower in parallel with the process towers. The synthesis planning and its execution using this robotically reconfigurable flow chemistry platform was illustrated by the synthesis of 15 drug and drug-like molecules.

Although the applications of robots for sample processing in flow analysis were already presented in the early 1990s, this method of enhancement of flow analytical systems did not gain much support in subsequent decades. This can be ascribed to the quite common perception of the instrumentation for flow analysis as a cost-effective way to improve laboratory operations; hence, adding robots to such set-ups could be too costly. A completely different story is the case of flow synthesis systems which are most beneficial for the pharmaceutical chemistry. The robotization of the searching process for new active pharmaceutical ingredients and the optimization of their syntheses has to be considered as an exceptionally correct and valuable choice in the further development of instrumentation for such needs.

An increasing trend in the construction of new devices and measuring systems, as well as in chemical analysis, is the hyphenation of conventional devices with mobile consumer electronics (e.g., smartphones or web cameras), as well as their use for the transmission of measurement data of global teleinformatic networks [330,331]. Mobile phones and smartphones are more and more commonly used as signal transducers for optical sensors and biosensors, as well as for the same purpose in the development of microfluidic devices with spectrophotometric or luminescence detections in recent years. For instance, a microfluidic-based smartphone dongle with colorimetric detection was developed for simultaneous assays of hemoglobin and human immunodeficiency virus (HIV) antibodies [332], while a wearable, cotton–thread–paper-based microfluidic device was also designed for colorimetric enzymatic glucose sensing in sweat [333]. A fluorescence immunoassay of *Escherichia coli* was adapted to a microfluidic format with smartphone signal transducing for the determination of that pathogen in urine [334], and electrochemiluminescence detection was employed in a paper-based microfluidic system with wax screen-printing channels and carbon ink-based screen-printed electrodes for the determination of hydrogen peroxide, which can also be adapted for point-of-care detections of glucose [335].

**Figure 8:**
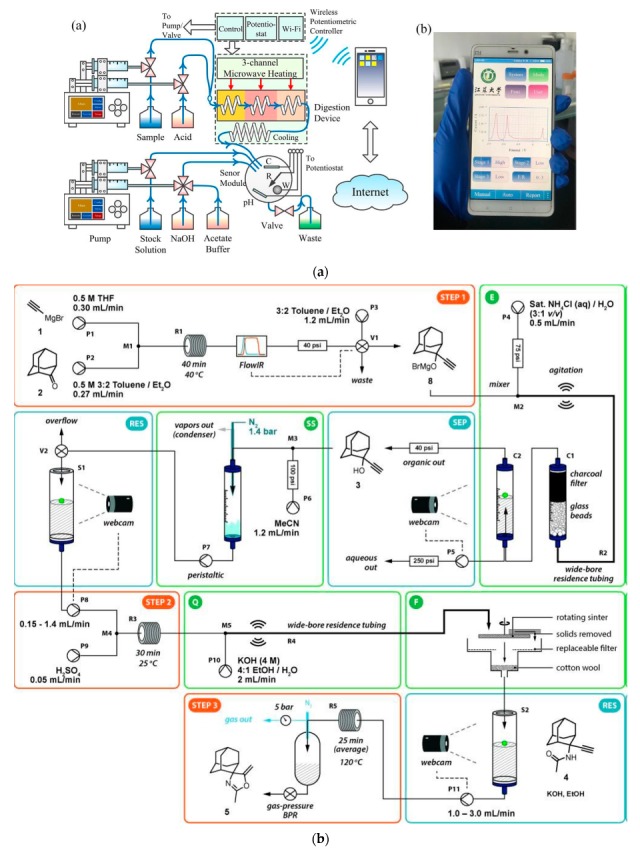
Examples of applications of webcam cameras in flow systems for analytical (**a**) [339] and synthetic (**b**) [340] applications: (**a**) manifold of measuring flow system involving programmable solid-state microwave flow digestion (a) and typical user interface (b) of the smart-phone-based electrochemical platform developed for determination of heavy metals in liquid foods; (**b**) seven-operation integrated flow platform for the synthesis of 2-aminoadamantane-2-carboxylic acid employing webcam cameras (P—pump; V—valve; M—mixer; R—reactor; C—column; S—reservoir). The output from the initial Grignard step is subjected to in-line quenching and then computer-controlled liquid–liquid phase separation. This solution undergoes a solvent switch, and the output is stored in a reservoir before being used for the Ritter reaction stage. The acidic output is quenched with a base, and the resulting salts are removed using a continuous filter. The filtrate is stored in a second reservoir before finally being heated to undergo cyclization.

Separate digital web cameras can also be employed for that purpose, replacing conventional spectrophotometers or luminescence detectors. This was illustrated, for instance, by the FIA lab-on-chip miniaturized system developed with the use of a photometric detector for acidity detection [336]. The fluorometric detection employing a web camera was reported in a conventional flow-batch system for the determination of a drug known as acetylcysteine with the use of Cd–Te quantum dots [337]. Smartphones or webcams can also be used in flow instrumentation to control the same functions, in addition to transducing a detection signal. This was reported, for instance, in a microfluidic set-up, where, in addition to the read-out biosensor, a smartphone was a part of the microfluidic liquid handling system combining elastomeric on-chip valves and compact pneumatic liquid pumping [338]. This can be applied in a bead-based fluoroimmunoassay, PCR-based analysis, flow cytometry, and nucleic acid sequencing. A similar concept was introduced into a smartphone electrochemical set-up developed for the determination of selected heavy metals in milk and fruit juices [339] (Figure 8a). In this case, the smartphone controls a programmable, prototype solid-state microwave flow digestion through a digital interface circuit, and it also performs fluidic operations (using a pump/valve) and differential pulse stripping voltammetry.

The use of web cameras was also reported in controlling certain fluidic operations in flow systems for the synthesis of organic compounds [341]. They involve, among others, the monitoring of chemical events in microdroplets, observing the reaction within the microwave cavity, the formation of aggregate deposits in the glass chip, recording crystallization processes, and the visualization of the liquid/liquid extraction process with the use of a plastic float, e.g., to adjust the flow rate of pumped phases. An exemplary application of three webcams in a self-controlling, telescoped seven-step synthesis was shown in a process including Grignard, Ritter, and cyclization reactions, where extractions, solvent-switching, filtration, and quenching steps were involved [340] (Figure 8b). The functioning of such a complex system can be controlled by only one operator.

In scientific and technical literature since the 1960s, there can be found various attempts at using telecommunication for medical purposes in order to facilitate long-distance doctor–patient interactions, as well as for environmental purposes, such as the remote monitoring of instrumen- tation and collected data transmission. Based on the tremendous progress made over the recent decades in personal computing and mobile telephony, it is not surprising to also find some examples of the application of modern telemetry in flow chemistry instrumentation. For instance, employing a computer sound card and a transmission wireless microphone, wireless recording of outputs from a spectrophotometer used for detection in the FIA system was reported for a distance up to 30 m [342]. Obviously, all the systems developed with data acquisition using mobile phones can be easily adapted for the same purpose. One more pioneering development in this field to be mentioned here was the application of camera phones for signal acquisition from paper-based microfluidic devices for low-cost off-site diagnosis [343]. This was demonstrated for carrying out the colorimetric determination of glucose and protein in urine. Then, in another approach employing a dedicated circuit plugged into the Universal Serial Bus (USB) port of a mobile phone, amperometric detection could also be carried out in a microfluidic system. This was reported for the determination of a protein, which is a biomarker of the malaria parasite, using a poly(dimethylsiloxane) microfluidic chip [344].

One more important aspect of the current developments of flow chemistry is worth mentioning here. In the last two decades, there was an increase in the associations of flow chemistry with the fast development of nanotechnology. In the field of flow analysis, this can be seen as an increasing interest in the application of two- and three-dimensional nanostructures for the enhancement of detection techniques, in separation processes carried out under flow conditions and in the creation of nanofluidic and microfluidic analytical systems [345]. In the development of new methods for spectral detections, the use of quantum dots proved to be particularly fruitful [346], while, in the development of electrochemical detections and separation methods, other inorganic nanoparticles with different functionalization were used [347]. Then, the development of especially efficient and fast methods for the synthesis of various nanoparticles was the subject of numerous works in the field of flow synthesis. These initially involved inorganic nanoparticles [348,349,350], where metallic nanoparticles found numerous catalytic applications in microflow reactors for organic syntheses [348]. Various nanoparticles and microparticles were synthesized under flow conditions [351], and functionalized nanoparticles [352] were designated for biomedical applications. Using microfluidic systems for this purpose enables precise control of the processes, as well as their significant shortening via appropriate modulation of the most critical stages. This leads to an improvement in the reproducibility of synthetic processes and a better control of the morphology and the size distribution of nanoparticles [352].

## 5. Conclusions and Perspectives

Flow chemistry is doubtlessly a very significant field in modern chemistry, with respect to conducting scientific research and various kinds of applications, moving from traditional appro- aches into inorganic, organic, and physical chemistry and chemical technology. In addition to the specialized books listed below, there were tens if not hundreds of reviews published, so far, on the different areas of flow chemistry discussed above, of which many were cited. Very recent reviews in the field of flow synthesis include a general review on flow syntheses as green methods of chemical syntheses [353], a presentation of equipment and separation units [199], an outline of the use of flow methods for taming hazardous chemistry [128], a description of the applications of flow micro- reactors and electrolysis cells in electrosynthetic processes [228,229], and a review on the pharmaceutical applications of flow synthesis [154]. In the field of flow analysis, general reviews on its recent advances [14] and 60-year development, based on publications in the analytical journal *Talanta* [354], can be mentioned. Furthermore, there were also reviews on the spectroanalytical applications of multi-syringe flow-injection methods [355], as well as on the application of fluidized particles in flow analysis methods [356], microfluidic paper-based analytical devices [262], or the use of flow analysis for the determination of radionuclides in nuclear industry [357]. Among the recent reviews on the technological applications of flow methods, much attention was focused on photochemical methods [85] and solar photochemistry [224].

The invention of flow chemistry in the middle of the previous century has to be attributed to the creation of the concept of flow analysis, which, at that time, was a real breakthrough in the world of chemical analytical methods. It was employed, first of all, in medical diagnostics, very quickly expanding into the areas of environmental analysis, food chemistry, and industrial processes. The last years of the previous century brought a very essential transformation of its instrumental format to microfluidic systems, and a parallel phenomenon took place in the rapid increase in the interest in conducting chemical syntheses under flow conditions in organic chemistry. 

The first apogee of the interest in flow analysis and its real routine applications was in the 1960s/1970s. Then, a certain renaissance took place between 1980 and 1990 along with the invention of injection methodologies. However, there were no spectacular commercial successes in this field, which is why chromatographic and atomic absorption techniques are more widely employed in routine analytical laboratories since then. Then, a new impetus was again provided in the last two decades with the creation of microsystems with wide prospects in medical applications, particularly for the design of devices for patient personal use. In the case of flow synthesis, the strongest impetus for its development came, without a doubt, from the pharmaceutical industry, where the search for new drugs is a top priority, and where microflow synthetic methodology trumps any other kind of instrumentation.

The main goal of this review article was to demonstrate how unjustified and far from reality it is to limit the term *flow chemistry* to flow synthesis, as can be found in thousands of published research papers. In fact, this notion concerns publications even in the most prestigious chemical scientific journals or books published by worldwide leading scientific publishers. Virtually all the metho- dologies of conducting chemical reactions or utilizing different unit operations are the same in flow analysis and flow synthesis, whereas their earlier applications are usually reported in flow analysis. The only difference between these two main fields of flow chemistry is the wide application of cryogenic conditions in conducting numerous reactions in organic syntheses, which are basically not used in flow analysis. It is truly bewildering how hermetic the progress is in the development of instrumental systems and methodologies in analytical and synthetic flow systems. Considering these two fields, twice as many books on flow analysis were published so far (Table 1), as compared to the books on flow synthesis (Table 2). However, in books on flow analysis published after 2000, one cannot find any remark on flow syntheses, although there is vast literature on carrying out different on-line reactions in flow analytical systems for sample treatment or the derivatization of analyte(s). On the other hand, none of the books on flow chemistry or flow synthesis mention the decades of development of flow analytical systems. One can find only some remarks on the use of analytical devices as additional accessories installed within flow synthetic systems for real-time monitoring of the progress of conducted reactions.

With regard to the briefly overviewed developments of these methodologies and instrumen- tation, it seems to be fully justified to use the term flow chemistry to represent all other chemical processes carried out under flow conditions of the reacting mixture, a sample to be analyzed, and other media chemically transformed under flow conditions. Certain symptoms of a slight evolution toward such a situation can be noted in some recent papers on microfluidics. the outstanding dynamics of the development of these technologies is reflected by the long-standing publication of dedicated scientific journals and books (already about 50 to date), as well as the presence of numerous manufacturers of such systems on the market. In an increasing number of research papers reporting new achievements, one can find remarks on their potential applications, both for analytical and for synthetic purposes, which is now the reality.

There are several numbers worth mentioning at the end of this review. They are related to bibliographical or scientometric aspects of the discussed field of scientific research, based on the Institute for Scientific Information (ISI) Web of Science in all database modes, viewed on 25 January 2020. It shows 1505 publications when the key phrase “flow chemistry” is entered, with a rapid increase after 2007 and about 210 papers per year published in the last three years. Quite similar results can be found when searching for the “flow synthesis” phrase, with 1139 publications, and a fast increase in the number of publications since 2005, with about 160 papers per year published in the last three years. A search for the “flow analysis” phrase shows 21,804 publications, but this cannot be considered as adequate since it also includes papers on fluid mechanics, economy, or marketing issues. More appropriate seem to be the data obtained for the “flow-injection analysis” phrase, which, in fact, can be considered as the main stream of the development of flow analysis in recent decades. The search for this entry shows 13,649 published papers, with about 250 papers published per year in the last three years. Such statistics clearly show the large activity in all considered fields; however, it must be pointed out that the search for the “microfluidic” phrase gives 72,585 papers published since 1989, with about 7000 papers per year published in the last three years. This seems to express the most important trend in this field, showing an equal importance of the future development of both flow analysis and flow synthesis.

## Figures and Tables

**Table 1:** Books published in English on flow analysis.

Title	Author(s)	Publisher
Continuous Analysis of Chemical Process Systems	S. Siggia	Wiley, 1959
Continuous-Flow Analysis. Theory and Practice	W.B. Furman	Marcel Dekker, 1976
Automatic Chemical Analysis	J.K. Foreman, P.B. Stockwell	Ellis Horwood, 1975
Automated Stream Analysis for Process Control	D.P. Manka	Academic Press, 1982
Flow-Injection Analysis	J. Ruzicka, E.H. Hansen	Wiley, 1^st^ Edition 1981, 2^nd^ Edition, 1988
Flow-Injection Analysis, Principles, and Applications	M. Valcarcel, M.D. Luque de Castro	Ellis Horwood, 1987
Flow-Injection Atomic Spectroscopy	J.L. Burguera (Ed.)	Marcel Dekker, 1989
Flow-Injection Analysis. A Practical Guide	B. Karlberg, G.E. Pacey	Elsevier, 1989
Flow-Injection Separation and Preconcentration	Z.-L. Fang	VCH Verlag, 1993
Flow-Injection Atomic Spectrometry	Z.-L. Fang	Wiley, 1994
Flow-Injection Analysis of Pharmaceuticals: Automation in the Laboratory	J.M. Calatayud	Taylor and Francis, 1997
Flow Analysis with Spectrometric Detectors	A. Sanz-Medel (Ed.),	Elsevier, 1999
Flow-Injection Analysis. Instrumentation and Applications	M. Trojanowicz	World Scientific, 2000
Advances in Flow Analysis	M. Trojanowicz (Ed.)	Wiley-VCH, 2008
Advances in Flow-Injection Analysis and Related Techniques	S.D. Kolev, I.D. McKelvie (Eds.)	Elsevier, 2008
Flow-Injection Analysis of Marine Samples	M.C. Yebra-Biurrun	Nova Science Publishers, 2009
Flow Analysis with Spectrophotometric and Luminometric Detection	E.A.G. Zagatto, C.C. Oliveira, A. Townshend, P. Worsfold	Elsevier, 2011
Flow Analysis: A Practical Guide	V. Cerda, L. Ferrer, J. Avivar, A. Cerda	Elsevier, 2014
Flow-Injection Analysis of Food Additives	C. Ruiz-Capillas, L.M.L. Nollet (Eds.)	CRC Press, 2015.
Flow and Capillary Electrophoretic Analysis	P. Kościelniak, M. Trojanowicz (Eds.)	Nova Science Publishers, 2017

**Table 2:** Books published on flow synthesis.

Title	Author(s)	Publisher
Chemical reactions and processes under flow conditions	S.V.Luis, E. Garcia-Varga	RSC, 2010
Micro reaction technology in organic synthesis	C. Viles, P. Watts	CRC Press, 2011
Flow chemistry. Fundamentals	F. Darvas, V. Hessel, G. Dorman	De Gruyter, 2014
Organometallic flow chemistry	T. Noël (Ed.)	Springer, 2016
Continuous-flow chemistry in the research laboratory	T. Glasnov	Springer, 2016
Sustainable flow chemistry: Methods and Applications	L. Vaccaro (Ed.)	Wiley, 2016
Flow chemistry for the synthesis of heterocycles	U.K. Sharma, E.V. Van der Eycken (Eds.)	Springer, 2018
Science of synthesis: Flow chemistry in organic synthesis	T.F. Jamison, G. Koch (Eds.)	Thieme,2018
Flow chemistry: Integrated approaches for practical applications	S.V. Luis, E. Garcia-Verdugo	RSC, 2019
